# Structural plasticity of the living kinetochore

**DOI:** 10.1083/jcb.201703152

**Published:** 2017-11-06

**Authors:** Karthik Dhatchinamoorthy, Manjunatha Shivaraju, Jeffrey J. Lange, Boris Rubinstein, Jay R. Unruh, Brian D. Slaughter, Jennifer L. Gerton

**Affiliations:** 1 Stowers Institute for Medical Research, Kansas City, MO; 2 The Open University, Milton Keynes, England, UK; 3 Department of Biochemistry and Molecular Biology, University of Kansas Medical Center, Kansas City, KS

## Abstract

The kinetochore is a large, evolutionarily conserved protein structure that connects chromosomes with microtubules. During chromosome segregation, outer kinetochore components track depolymerizing ends of microtubules to facilitate the separation of chromosomes into two cells. In budding yeast, each chromosome has a point centromere upon which a single kinetochore is built, which attaches to a single microtubule. This defined architecture facilitates quantitative examination of kinetochores during the cell cycle. Using three independent measures—calibrated imaging, FRAP, and photoconversion—we find that the Dam1 submodule is unchanged during anaphase, whereas MIND and Ndc80 submodules add copies to form an “anaphase configuration” kinetochore. Microtubule depolymerization and kinesin-related motors contribute to copy addition. Mathematical simulations indicate that the addition of microtubule attachments could facilitate tracking during rapid microtubule depolymerization. We speculate that the minimal kinetochore configuration, which exists from G1 through metaphase, allows for correction of misattachments. Our study provides insight into dynamics and plasticity of the kinetochore structure during chromosome segregation in living cells.

## Abstract (precis)

Dhatchinamoorthy et al. use calibrated imaging, FRAP, and photoconversion to study the changes in kinetochore component copy numbers from G1 to anaphase and find that the Dam1 submodule is unchanged during anaphase, whereas MIND and Ndc80 submodules add copies, providing insight into the dynamics and plasticity of the kinetochore structure during chromosome segregation.

## Introduction

Pairs of sister chromatids must be precisely divided into two cells during cell division to prevent missegregation. Chromosome missegregation results in aneuploidy, which is associated with cancer and birth defects (Yuen et al., 2005; Pfau and Amon, 2012). Therefore, understanding the mechanisms of chromosome segregation is critical to understanding the fidelity of chromosome transmission. Microtubules attach to the chromosome via kinetochores and pull them to the poles during chromosome segregation. The kinetochore is a several-megadalton–sized protein structure assembled on a specialized region of the chromosome called the centromere. The centromeric region is a ∼130-bp sequence in budding yeast and is epigenetically defined in higher organisms. Most of the kinetochore proteins and their functions are evolutionarily conserved. In higher eukaryotes, each kinetochore interacts with multiple microtubules (McAinsh et al., 2003; Chan et al., 2005; Walczak et al., 2010), whereas in *Saccharomyces cerevisiae*, each chromosome interacts with only one microtubule (Winey et al., 1995), making it an ideal defined system for studying kinetochore–microtubule interaction.

**Figure 1.**
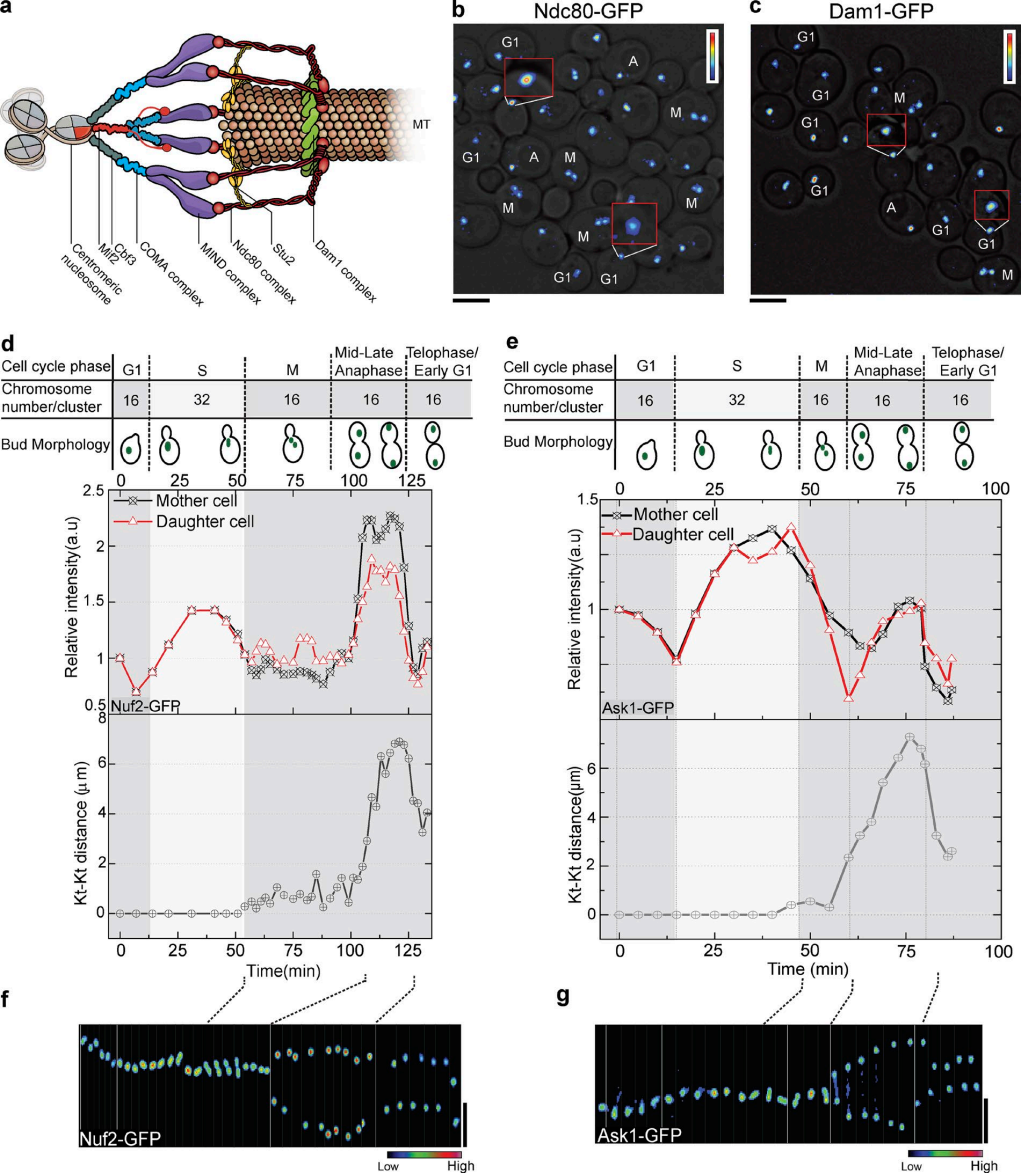
**The intensity of some kinetochore submodules increases in anaphase.** (a) Model of a yeast kinetochore (Kt) showing inner and outer kinetochore complexes. The Ndc80 complex interacts with microtubules (MTs) with fingerlike projection; the Dam1 complex can form a ringlike structure. (b and c) Heat map analyses of Ndc80-EGFP (b) and Dam1-EGFP (c) intensities in an asynchronous culture. Cell cycle stages have been marked near the cell. Red inserts show enlarged intensity heat map. Ndc80 is much brighter in anaphase than G1. The intensity of Dam1 is similar in G1 and anaphase. (d and e) Intensity profiles of Nuf2-EGFP (d) and Ask1-EGFP (e) over the cell cycle are shown. The distance between the two kinetochore clusters (Kt-Kt distance, bottom graphs) helps to define the cell cycle stage in addition to the bud morphology (top). (f) Kymograph of Nuf2-EGFP heat map over the cell cycle shows an increase in intensity as kinetochore clusters separate in anaphase. (g) Kymograph of Ask1-EGFP heat map shows G1 and anaphase kinetochore clusters have similar intensity. Bars, 5 µm.

The yeast kinetochore (Fig. 1 a) consists of >60 proteins that assemble into submodules, constituting the inner and outer kinetochore. The inner kinetochore consists of CBF3–Ndc10, Mif2, COMA, and Cnn1 complexes that connect the centromeric region of the chromosome to the outer kinetochore. The MIND–Mis12, Spc105–CeKNL-1, Dam1, and Ndc80 complexes form the outer kinetochore and link the inner kinetochore to the microtubule (De Wulf et al., 2003; Lampert and Westermann, 2011). The Ndc80 complex is a heterotetramer of Spc25, Spc24, Nuf2, and Ndc80. Ndc80 binds to the tubulin subunits of a microtubule using an unstructured N terminus as a fingerlike projection along with the calponin homology domain (Guimaraes et al., 2008; Powers et al., 2009). The MIND–MIS12 complex, a heterotetramer of Nsl1, Nnf1, Dsn1, and Mtw1, links the Ndc80 complex to the inner kinetochore (Obuse et al., 2004; Hornung et al., 2011). The Dam1 complex, a heterodecamer, is the functional analogue of the Ska1 complex in higher eukaryotes and forms a ringlike structure around the microtubule in vitro that may slide along the microtubule as it disassembles (Westermann et al., 2006; Wang et al., 2007; Welburn et al., 2009; Schmidt et al., 2012; van Hooff et al., 2017). Ndc80 and Dam1 complexes use different means of microtubule contact but work cooperatively to track the disassembling end of the microtubule during chromosome segregation (Powers et al., 2009; Tien et al., 2010; Umbreit et al., 2014), resulting in the movement of chromosomes to the poles (Grishchuk and McIntosh, 2006). Although some structural details of the kinetochore submodules that provide attachments to microtubules have been determined, how the submodular structure for kinetochore–microtubule attachments functions in a living cell is not clear.

A microtubule is a polymer of α and β tubulin dimers and interacts with the outer kinetochore to pull the chromosome. A microtubule is a highly dynamic structure, as it can add or lose tubulin dimers in processes called “rescue” and “catastrophe,” respectively (Desai and Mitchison, 1997). Growing and shrinking microtubule tip structures differ by their degree of microtubule protofilament curve, which can influence the interaction with the Ndc80 complex (Asbury et al., 2011; Foley and Kapoor, 2013). Microtubule-associated proteins (MAPs) can alter the microtubule dynamics by interacting with the microtubule tip and the tubulin dimers. MAPs have been implicated in chromosome capture, spindle stability, and chromosome movement (Pearson et al., 2003; Gandhi et al., 2011). Stu2, one of the XMAP215 family proteins, specifically interacts with the Ndc80 complex (Miller et al., 2016) and affects microtubule behavior. Loss of Stu2 results in loss of tension, leading to missegregation of chromosomes in the absence of the spindle assembly checkpoint (Miller et al., 2016). Although the function of Stu2 has been studied in metaphase with purified kinetochores, the function of MAPs during anaphase, when a kinetochore must track a microtubule with prolonged rapid disassembly, has not been examined.

We present data consistent with structural plasticity of the kinetochore during chromosome segregation. Our calibrated imaging, FRAP, photoconversion, and genetic studies suggest that the outer-kinetochore complexes—especially the MIND and Ndc80 complexes—add new copies of proteins during anaphase. However, the Dam1 complex remains unchanged. Both the MAP Stu2 and the kinesin motor Kar3, working with Vik1, facilitate the copy number increase of MIND and Ndc80 subcomplexes in anaphase, suggesting these kinetochore submodules can adjust their addition based on the rate of microtubule depolymerization. Simulations of kinetochore function using Hill’s kinetochore attachment model (Hill, 1985) predict that the addition of each coupler (or copy) decreases the detachment rate by approximately fourfold, suggesting addition of the Ndc80 submodule could improve attachment during anaphase. We observe similar structural changes in kinetochores in fission yeast, suggesting structural plasticity is an evolutionarily conserved property of kinetochores in higher eukaryotes. Collectively, our results suggest that parts of the kinetochore structure can sense and adapt to microtubule dynamics, whereas other parts remain constant. Overall, the design of the kinetochore may accommodate structural plasticity that promotes accurate segregation of chromosomes.

## Results

### Submodules of the yeast kinetochore increase in intensity during anaphase

Budding yeast has 16 chromosomes with centromeres that form a cluster (Jin et al., 1998, 2000), and EGFP-tagged kinetochore proteins form a subdiffraction-limited spot (Joglekar et al., 2008b). Kinetochores form a single cluster from G1 through late S phase. When sister kinetochores biorient and start to separate, the single cluster divides into two equal clusters with half as many kinetochores. To investigate the kinetochore structure during the cell cycle, we used endogenously expressed kinetochore proteins tagged with EGFP at the C terminus to examine fluorescence intensity. In an asynchronous culture, cells having no bud were categorized as G1 phase, whereas anaphase cells exhibited the maximum distance between kinetochore clusters or spindle pole bodies (SPBs). The fluorescence intensity of kinetochore clusters, represented by a heat map, showed that subunits of the Ndc80 complex had higher intensity in anaphase cells than in G1 cells (Fig. 1 b). However, Dam1p of the Dam1 complex had similar intensity in both anaphase and G1 cells (Fig. 1 c).

We further quantified the intensity of the kinetochore cluster through the cell cycle by arresting cells in G1 using α factor and subsequently releasing them for imaging. As expected, the intensity of the Nuf2 (Ndc80 complex) cluster increased during S phase based on the doubling of kinetochore structures (16 to 32) and then dropped as the cell progressed through metaphase, and the 32 kinetochores split into two groups of 16 (Fig. 1 d). An exact doubling of intensity is not always seen in S phase because of difficulties in measurement of the cluster during this stage (Shivaraju et al., 2012). The fluorescence intensity of the cluster increased as the chromosomes separated during anaphase (Figs. 1 d and S1 a). At the end of anaphase, the fluorescence intensity of the 16-chromosome cluster was nearly twofold higher than G1, suggesting more proteins were present in each kinetochore structure (Fig. 1 d). We examined all proteins from the Ndc80 and MIND complexes (not depicted), finding similar intensity profiles to Nuf2 and Dsn1 (MIND complex; Fig. S1, b and c). In contrast, similar experiments with Dam1 complex proteins showed little intensity increase during anaphase relative to G1. As an example, we showed Ask1, the intensity of which increased from G1 to S phase as expected but did not increase during anaphase (Figs. 1 e and S1 d). A kymograph heat map analysis showed that Nuf2 increased in intensity during anaphase, whereas Ask1 did not (Fig. 1, f and g). Our analyses indicate that the increase was specific to anaphase because the intensity dropped in the subsequent G1 stage.

### Some kinetochore subcomplexes increase during anaphase

To further quantify the fluorescence increase, we used calibrated imaging, which is based on measuring the absolute intensity of EGFP in live yeast using fluorescence correlation spectroscopy (FCS). The intensity of a single EGFP is determined each time an experiment is performed, and this value is used to determine copy number based on brightness of the kinetochore cluster (Smith et al., 2014). Nuclear pore complex proteins have been used extensively to validate this method (Shivaraju et al., 2012). Furthermore, calibrated imaging was used to demonstrate that the copy number of Cse4-GFP in diploid cells is twice that of haploid cells, strengthening confidence in the accuracy of the method (Shivaraju et al., 2012). We collected multiple measurements on multiple days to identify and verify strong trends.

Strains with EGFP epitope–tagged proteins of Ndc10, Ctf19/COMA (hereafter COMA), Cnn1, Spc105, MIND, Ndc80, and Dam1 kinetochore complexes were used for quantification in G1 and anaphase. All strains were karyotyped to validate normal ploidy (see the Yeast strains section of Materials and methods; Table S1). Although data were collected for calibrated imaging from an asynchronously growing culture, we compared calibrated imaging results for Dsn1 during G1 using an asynchronous culture-versus–α-factor arrest (Fig. S2 a). The copy number was independent of the method used to obtain G1 cells. These data also demonstrate the reproducibility of the calibrated imaging method between two independent experiments.

**Figure 2.**
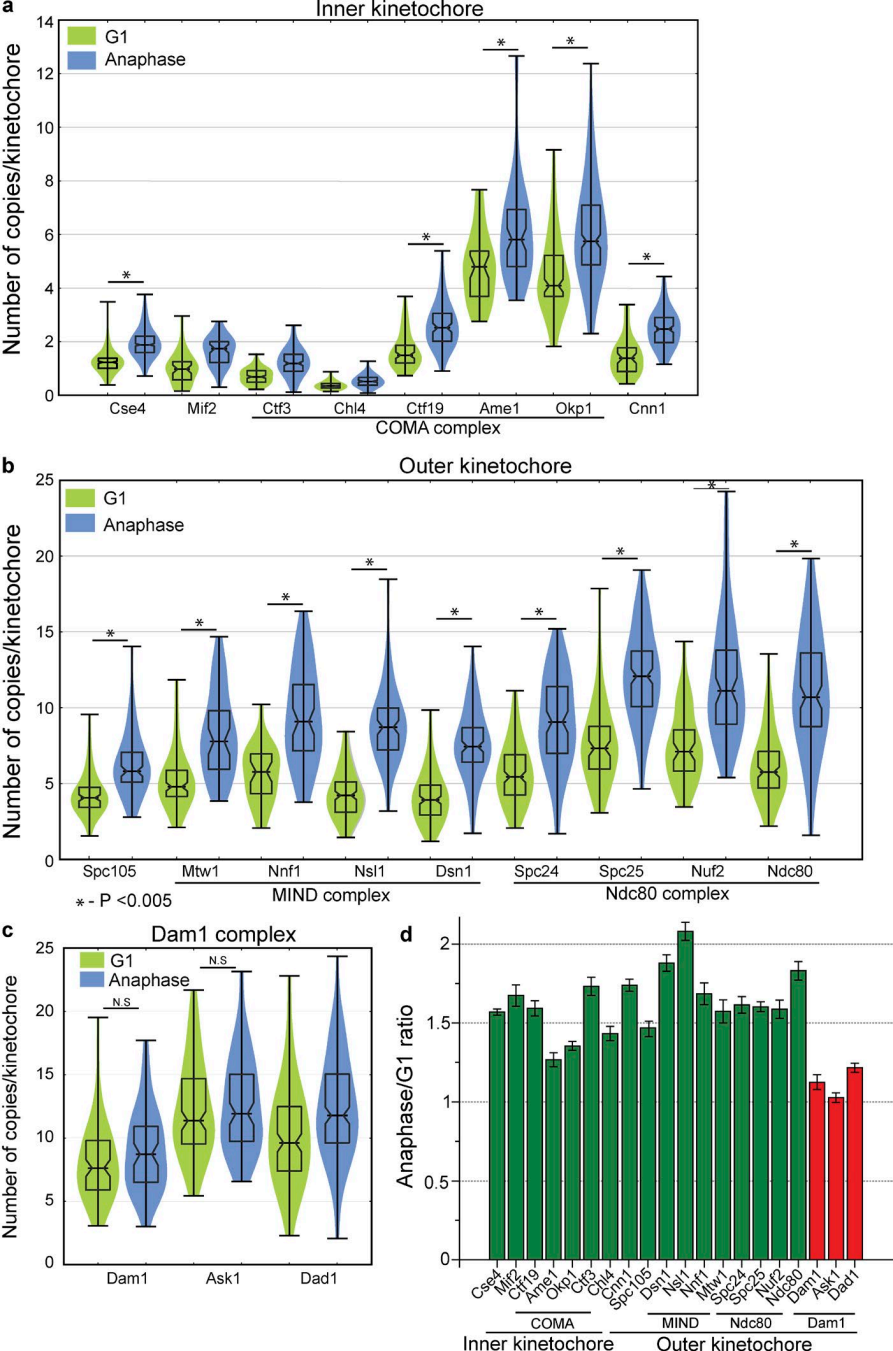
**The kinetochore has higher copy numbers of Ndc80 and MIND subcomplexes in anaphase, whereas the Dam1 complex has similar copy number in G1 and anaphase.** (a) Violin plot of copy numbers of the inner kinetochore proteins in G1 and anaphase. The horizontal middle line is the mean, and the box represents the SD. 50–173 clusters were used to calculate the copy number. (b) Violin plot showing copy number of the outer kinetochore proteins. Subunits in the MIND and Ndc80 subcomplexes nearly double in copy number in anaphase. 50–150 clusters were used to calculate the copy number. (c) Violin plot showing the copy number of the subunits of the Dam1 subcomplex with similar counts in G1 and anaphase. 50–280 clusters were used to calculate the copy number. For a–c, a two-tailed *t* test was used to test for statistical significance. (d) The ratio of kinetochore proteins in anaphase to G1 (*n* = 3) show that the subunits of the MIND and Ndc80 subcomplexes have higher copy number in anaphase as compared with the Dam1 subunits. Error bars represent SEM.

Several of the inner kinetochore proteins displayed a significant increase in their copy number during anaphase (Fig. 2 a). Outer kinetochore proteins also displayed an increase in copies during anaphase (Fig. 2 b). These included proteins from the MIND and Ndc80 complexes, which increased in copy number in anaphase by 50–100% (Figs. 2 d and S2 b). Subunits of the COMA subcomplex, such as Okp1 and Ame1, also increased in copy number in anaphase (Fig. 2 a). The MIND complex is joined to the inner kinetochore by COMA (Hornung et al., 2014) and associates with the microtubule-associated Ndc80 complex of the outer kinetochore. These three subcomplexes all displayed increases as chromosomes are separating during anaphase, consistent with the idea that they operate together structurally.

The subunits of the Dam1 complex, in contrast to subunits of COMA, MIND, and Ndc80 complexes, had a similar copy number in G1 and anaphase (Fig. 2, c and d). Subunits of the Dam1 complex show only a slight increase in copy number between G1 and anaphase (Fig. 2 c). The mean distribution of 12 ± 4 (SEM) correlates reasonably well with the modeling of 16-fold symmetry of a Dam1 ring encircling a microtubule assembled in vitro (Westermann et al., 2006; Wang et al., 2007). Overall, inner-complex proteins tended to be present at lower copy numbers relative to the outer-complex proteins, consistent with a structure with an initial anchoring point to the centromere with amplification moving toward the microtubule side.

Debate in the past over calibrated imaging of centromere/kinetochore proteins in yeast has centered on the size of the cluster, which undergoes compaction in anaphase (Joglekar et al., 2006). Simulations of centromere compaction demonstrated an observed increase in the peak intensity of the cluster of centromeric Cse4 by ∼40% (Aravamudhan et al., 2013). It is worth noting that the 40% estimate (Aravamudhan et al., 2013) for differences in amplitude assumed a 1.4-NA objective, and thus is inaccurate when applied to the data presented in this study and in a previous publication (Shivaraju et al., 2012), as the current data were acquired with a 1.2-NA objective. The difference in amplitude expected based on spot size is highly dependent on the resolution of the system, and given our resolution, the maximum difference in amplitude we could expect based on compaction was 20–25% (Smith et al., 2014). We took specific steps to ensure that the data presented in this study are not clouded by this complication. First, we included data only on spots whose fit width was under a threshold (set as an SD of 182 nm for the Gaussian fit; Shivaraju et al., 2012). The increase by ≥50% by the end of anaphase for many of the kinetochore proteins after selecting similarly sized clusters surpassed the 25% increase that may occur as a result of compaction. Furthermore, we did not observe an increase in the Dam1 complex despite the similar extent of compaction observed for it relative to other kinetochore submodules. These factors strongly suggest that the intensity increase we observed was not caused by the compaction of the kinetochore.

Nonetheless, we repeated our analysis with the integrated intensity method (Joglekar et al., 2006), which is immune to artifacts from compaction. After applying the same-sized filter as above to eliminate spots that were disperse, we integrated the total intensity of each spot in G1 and anaphase. We normalized the kinetochore protein intensities to the mean integrated intensity of Cse4 during anaphase (which we set as 2 Cse4/kinetochore; Fig. S2 c). Although noise increased as expected based on integration, we obtained consistent results, with many proteins in the inner and outer kinetochore increasing in copy number in anaphase, whereas the Dam1 complex did not.

### MIND and Ndc80 complexes recover in FRAP, but Dam1 complex does not

**Figure 3.**
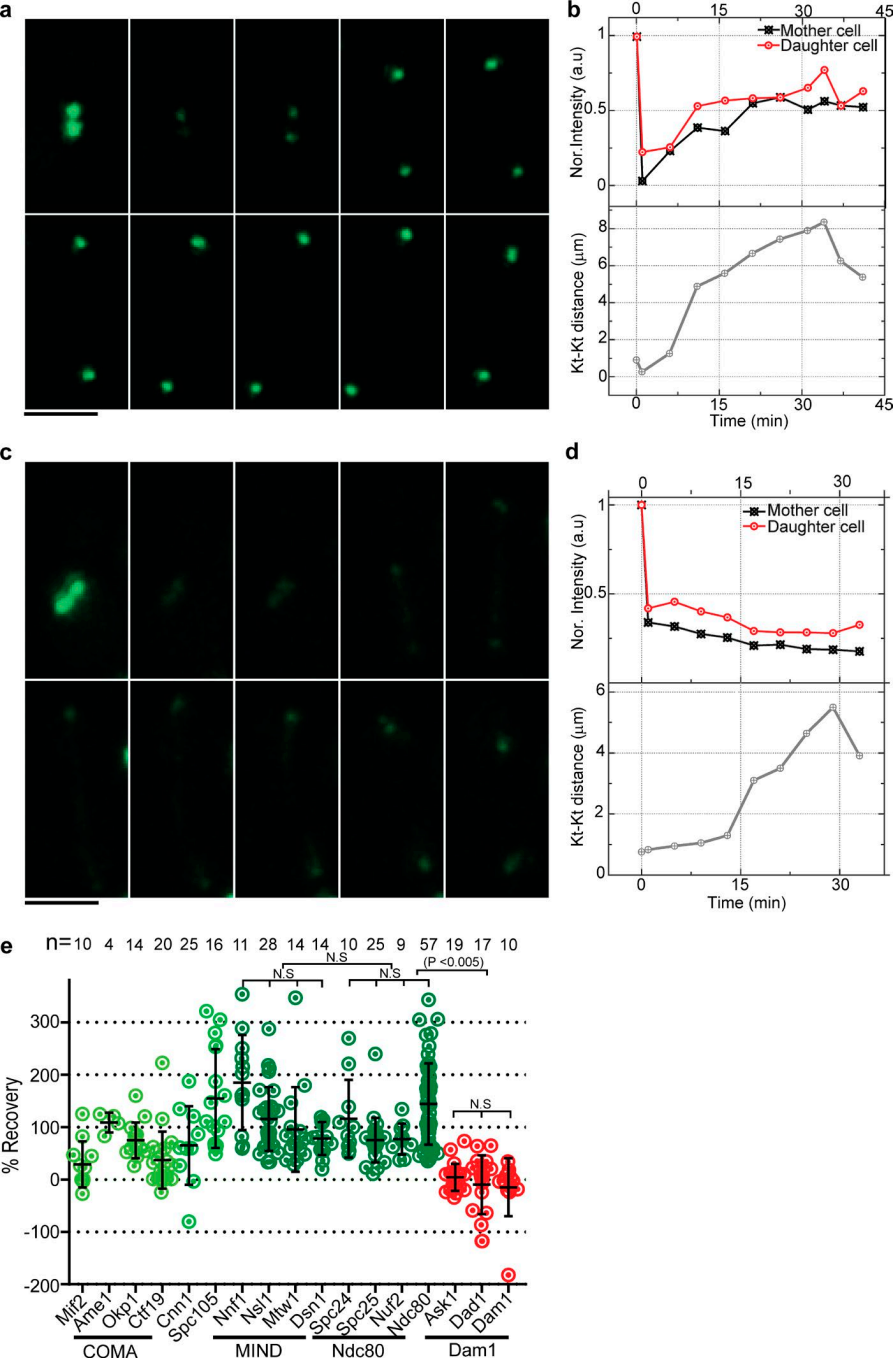
**Subunits of the Ndc80 and MIND subcomplexes recover in FRAP, whereas subunits of the Dam1 subcomplex do not.** (a and b) Kinetochore (Kt) clusters in metaphase with Nuf2-EGFP were bleached once they were separated by <2 µm (1 min), and recovery was monitored and the intensities were quantified from a single cell (*n* = 9). For both the mother and daughter clusters, intensity was normalized to 1 at 0 min. The second time point represents a photobleach step. (c and d) Kinetochore clusters with Ask1-EGFP were followed as in a and b. Quantification of the Ask1-EGFP kinetochore cluster intensity from a single cell (*n* = 19; d) shows no recovery. Bars, 5 µm. (e) Percent recovery after photobleaching for kinetochore proteins is shown; each dot represents an individual cluster. Subunits of the COMA (Ame1 and Okp1), MIND, and Ndc80 complexes show >80% recovery in anaphase (green), whereas Mif2 and subunits of the Dam1 subcomplex show little recovery (red). A two-tailed *t* test was used to test for statistical significance.

If copies of a kinetochore protein are added in anaphase, FRAP experiments should reveal this addition. We bleached the kinetochore cluster in metaphase (when kinetochore clusters are <1 µm apart) and followed it through the cell cycle to quantify the percent recovery in the subsequent anaphase. As the kinetochore clusters moved toward the pole, proteins from the COMA, MIND, and Ndc80 complexes recovered to varying degrees (Fig. 3, a and b; and Fig. S3, a–c). Quantification of subunits of the bleached MIND and Ndc80 complexes shows a recovery of >80% in late anaphase (Fig. 3 e). The recovery of a few subunits of the MIND and Ndc80 subcomplexes in FRAP beyond the starting value (>100%) suggests that these complexes might have more protein in anaphase than metaphase. However, Ask1 and other subunits of the Dam1 complex failed to recover in anaphase (Fig. 3, c and d; and Fig. S3 d). Although we saw some experimental variation of the percent recovery with subunits of the same complex, we attribute this in part to variable bleaching of the nucleoplasm. Furthermore, proteins with low starting copy number such as Mif2 were challenging to image without significant bleaching. Overall, however, our FRAP experiments revealed that all of the Ndc80 and MIND subunits examined recovered more fluorescence than the Dam1 subunits (Figs. 3 e and S3). Furthermore, the recovery in MIND, Ndc80, and Dam1 subunits was not statistically different within the complex, and MIND and Ndc80 were not statistically significantly different from each other, but both were statistically significantly different from Dam1 (P < 0.0005). Similar FRAP experiments performed on metaphase clusters by ourselves (not depicted) and others (Joglekar et al., 2008b; Suzuki et al., 2016) resulted in no recovery within metaphase, suggesting that recovery is specific to progression from metaphase through anaphase. Furthermore, bleaching in mid- to late anaphase did not result in recovery within anaphase as determined by ourselves (not depicted) and others (Joglekar et al., 2008b; Suzuki et al., 2016). Collectively, the FRAP data are consistent with the addition of MIND and Ndc80 subunits from early to late anaphase and little addition of Dam1 complex subunits.

### MIND and Ndc80 complexes add copies during anaphase, whereas the Dam1 complex remains stable

**Figure 4.**
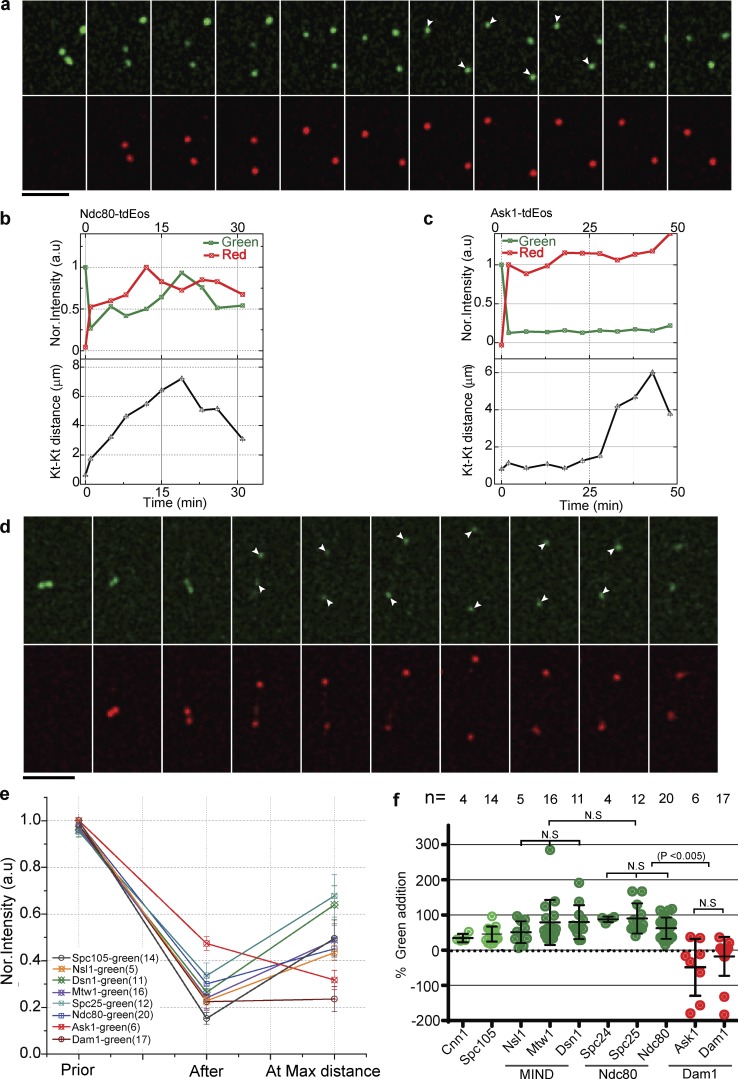
**Ndc80 and MIND subcomplexes add new copies during anaphase, whereas Dam1 does not.** (a) Ndc80-tdEos is photoconverted in metaphase (4 min) and followed through anaphase. The increase in green fluorescence (white arrowheads) suggests that new copies are added. (b) Quantification of normalized green and red fluorescence intensities at the Ndc80-tdEos spot is plotted over time from a single cell (*n* = 20). (c and d) A similar analysis was performed for Ask1-tdEos. The normalized green fluorescence plot shows that new protein is not added during anaphase. For a and d, the prephotoconverted images have been saturated for better visualization; please refer to the quantifications in b and c. Bars, 5 µm. (e) The mean change in the normalized green fluorescence intensity is shown for each kinetochore (Kt) protein during anaphase. Error bars represent SEM. The number of experiments is indicated in parentheses. (f) Graph showing the percentage of green fluorescence addition during anaphase by kinetochore protein for the number of individual clusters indicated. MIND and Ndc80 subcomplexes (green) have more green fluorescence at the maximum kinetochore distance than Dam1 subunits (red). A two-tailed *t* test was used to test for statistical significance.

Our FRAP studies indicate that submodules of the kinetochore, especially the MIND and Ndc80 complexes, are dynamic during anaphase. However, FRAP cannot distinguish between complete protein turnover versus retention of old subunits plus the addition of new subunits. To address this issue, we used kinetochore proteins tagged with a photoconvertable epitope (tdEos) that can be converted from green to red fluorescence with a brief exposure to ultraviolet light (405 nm; McKinney et al., 2009; Wisniewski et al., 2014). We selectively photoconverted the kinetochore cluster in metaphase and followed it through anaphase. Preexisting protein will be red after photoconversion, and protein in the nucleoplasm will remain green (with the exception of a small amount of off-axis photoconversion, which we estimated to be >5%). Photoconversion of the cluster was >70% in our experiments. We then monitored the addition of protein to the kinetochore during anaphase. Addition of green fluorescence was unmistakable evidence of the addition of protein subunits. In the case of Ndc80-tdEos, we observed a >40% increase in green intensity at the kinetochore cluster during anaphase, suggesting that new copies were being added (Fig. 4, a and b). Red fluorescence at the kinetochore cluster was similar throughout the experiment, implying that the preexisting protein was maintained, although in some experiments a slight increase in red fluorescence was observed, possibly because of photoconversion of nearby nucleoplasm (Fig. S4 a).

A similar photoconversion experiment using Ask1-tdEos from the Dam1 complex did not show any addition of new protein in anaphase (Fig. 4, c and d; and Fig. S4 e), suggesting this complex is more stable. Cnn1 and Spc105 were added during anaphase (Fig. S4 b). Photoconversion experiments suggest MIND and Ndc80 complexes added >50% new protein in anaphase (Fig. 4, e and f; and Fig. S4, c and d), whereas the Dam1 complex added little, if any, new protein in anaphase. The addition in MIND, Ndc80, and Dam1 subunits was not statistically different within the complex, and MIND and Ndc80 were not statistically significantly different from each other, but both were statistically significantly different from Dam1 (P < 0.0005).

**Figure 5.**
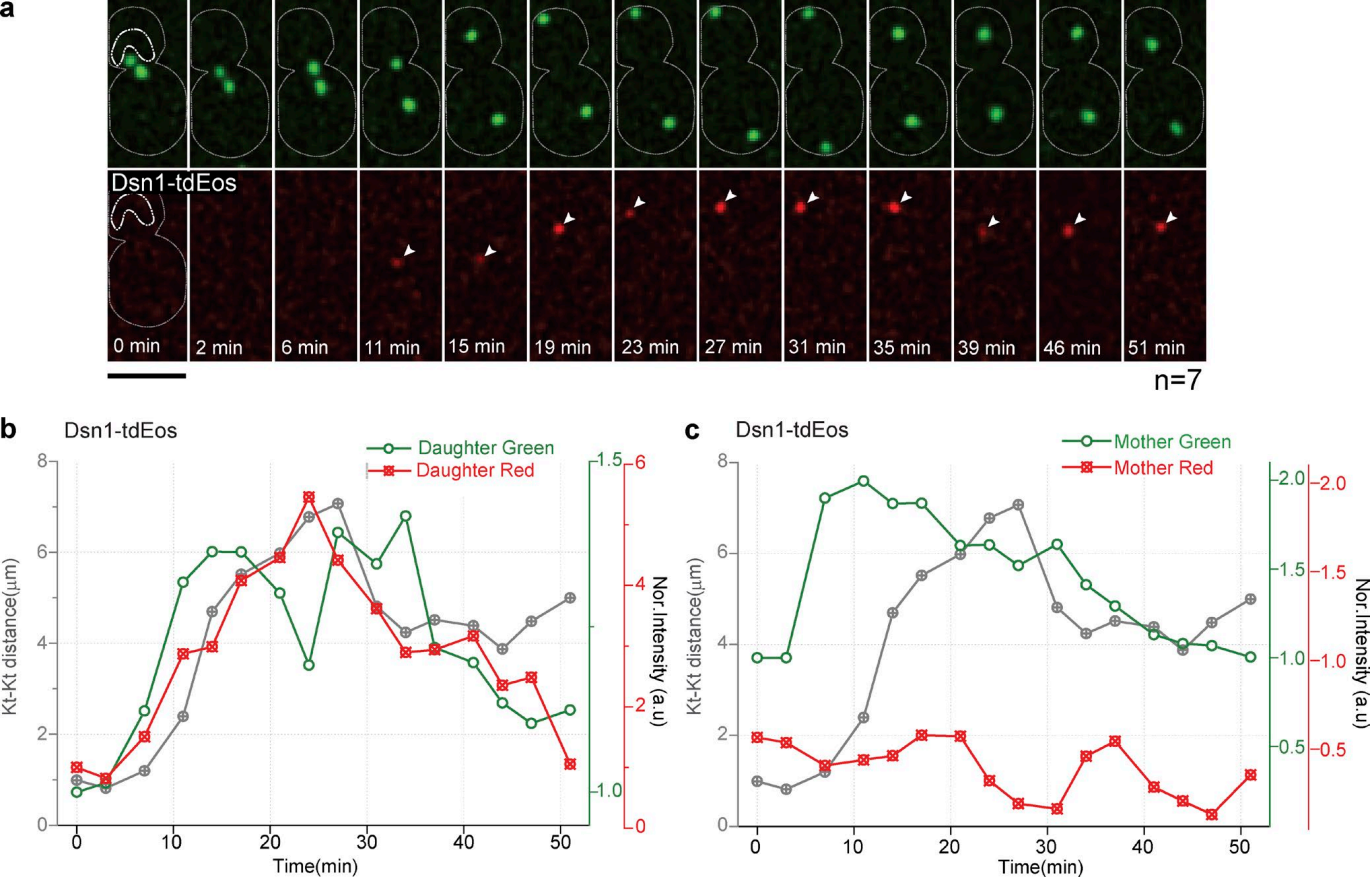
**Photoconversion of Dsn1-tdEos in the nucleoplasm reveals copy addition during anaphase.** (a and b) Snapshots with quantification of a nucleoplasm photoconverted (marked with a white dotted line) cell from metaphase through anaphase. Protein from photoconverted nucleoplasm (red) in the daughter cell (top cluster) was added to the kinetochore (Kt) during anaphase. The addition of new protein is demonstrated by the increase in the red fluorescence (white arrows). Bar, 5 µm. (c) The mother cell kinetochore (bottom cluster) without nucleoplasm photoconversion shows the expected increase in green fluorescence but no increase in red fluorescence during anaphase. This experiment was repeated seven times with similar results.

To rule out the possibility that the increase in green fluorescence might be caused by the compaction of the kinetochore or that folding dynamics of tdEos are a factor, we photoconverted the surrounding nucleoplasm during metaphase, changing the protein to its red form, without photoconverting the kinetochore cluster (leaving it green). As these cells progressed into anaphase, red fluorescence was added to the kinetochore cluster, strongly suggesting that new copies were added from the nucleoplasm (Fig. 5). Collectively, the independent observations from FRAP, photoconversion, and calibrated imaging experiments suggest that additional copies of the MIND and Ndc80 subcomplexes were added to kinetochores as cells progressed from metaphase to anaphase, while the Dam1 complex remained constant.

### MAPs affect kinetochore copy number

As the kinetochore tracks the depolymerizing microtubules during anaphase, chromosomes move to the poles. MAPs affect microtubule function and dynamics (Mallavarapu et al., 1999; Wolyniak et al., 2006). Bik1, Stu2, and Bim1 are MAPs that locate near the kinetochore–microtubule attachment site (He et al., 2001; Aravamudhan et al., 2014). Stu2/XMAP215 (*STU2* homologue in human) has been implicated as a microtubule destabilizer and in some cases as a microtubule polymerase (van Breugel et al., 2003; Al-Bassam et al., 2006; Podolski et al., 2014). Stu2 is important for spindle elongation and stabilization during anaphase (Kosco et al., 2001; Severin et al., 2001; van Breugel et al., 2003). Stu2 mutants have slower microtubule dynamics with a longer paused state and shorter spindles (He et al., 2001; Pearson et al., 2003). Ndc80 complexes physically interact with Stu2 (Miller et al., 2016). A yeast strain bearing a temperature-sensitive *stu2-11* allele has slower spindle elongation and takes longer to complete anaphase than WT cells (∼10 min slower). Mutations that affect MAPs can therefore affect microtubule dynamics.

**Figure 6.**
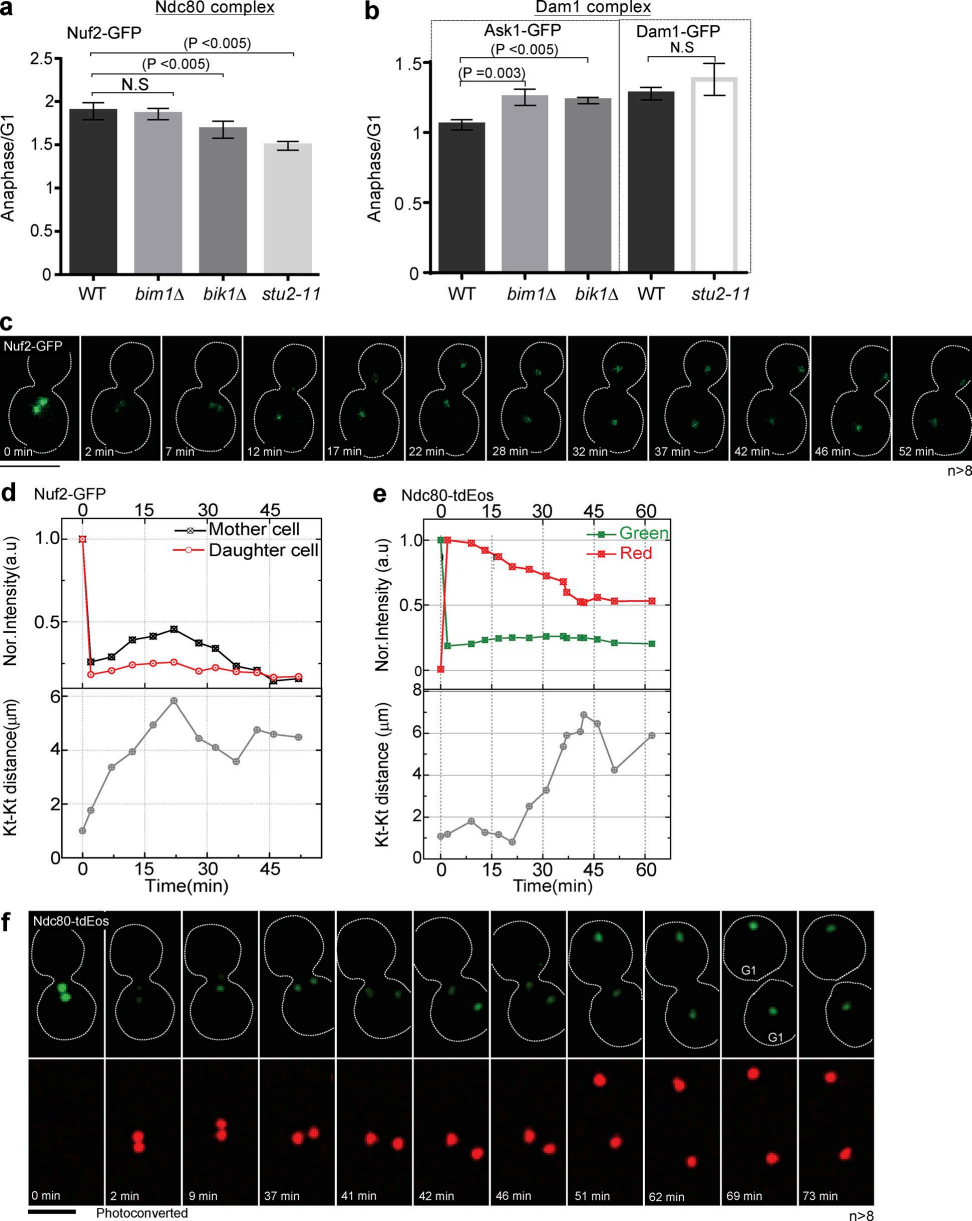
**A mutation in *STU2* reduces the addition of Ndc80 subunits in anaphase.** (a and b) Calibrated imaging was used to calculate the copy number for the protein indicated in each MAP mutant during G1 and anaphase, similar to Fig. 2, and then was plotted as in Fig. 2 d. The *stu2-11* mutant has a lower anaphase/G1 ratio than WT for Nuf2, whereas Dam1 is unaffected. The *bik1Δ* and *bim1Δ* mutants had a lower copy number for Ask1 in G1, affecting the anaphase/G1 ratio. 50–130 clusters were considered for the copy number calculation (*n* = 3). (c and d) Snapshots and quantification of FRAP from a single cell shows that Nuf2 does not recover during anaphase in the *stu2-11* background (*n* > 8). (e and f) Photoconversion of Ndc80-tdEos in the *stu2-11* background shows that green fluorescence does not increase in anaphase from a single cell (*n* > 8). Bars, 5 µm. Dotted white lines mark cell peripheries. All experiments were performed at room temperature. A two-tailed *t* test was used to test for statistical significance, and the error bars represent SEM. Kt, kinetochore.

To test the effect of microtubule dynamics on kinetochore structure, we used strains with compromised MAP function, deleting *BIK1* and *BIM1*, or using a temperature-sensitive allele of the essential *STU2*, *stu2-11*. Calibrated imaging revealed that the *stu2-11* mutant at room temperature (25°C) had fewer copies of the Ndc80 complex in anaphase compared with the WT strain or the *bim1*Δ or *bik1*Δ strains, suggesting microtubule dynamics controlled by Stu2 could affect anaphase addition (Fig. 6 a). In contrast, the Dam1 complex was relatively unaffected by compromising MAP function, suggesting that microtubule dynamics more specifically affect the Ndc80 complex (Fig. 5 b). FRAP experiments show poorer recovery of Nuf2-GFP in the *stu2-11* mutant compared with WT (Fig. 6, c and d). Furthermore, photoconversion experiments suggest Ndc80-tdEos failed to add copies during anaphase in the *stu2-11* strain (Fig. 6, e and f). Collectively, these data suggest that microtubule destabilization with slower spindle elongation in the *stu2-11* mutant specifically affects the number of Ndc80 complexes added during anaphase. We speculate that the addition of the Ndc80 submodule can adjust based on the rate of microtubule depolymerization during anaphase, whereas the Dam1 submodule structure is relatively immune to microtubule dynamics during anaphase.

### Addition of MIND and Ndc80 subcomplexes in anaphase is coupled to established cell cycle factors

Having established that the kinetochore is a plastic structure with a minimal copy number state or “G1 configuration” from G1 to metaphase and a high-copy number state or “anaphase configuration” in anaphase as well as that it may respond to microtubule depolymerization, we next asked how this structural transition is coupled to known regulators of metaphase and anaphase. We evaluated the addition of MIND and Ndc80 in many different mutant backgrounds, including mutations compromising (a) intersister centromere tension, (b) microtubule motors, (c) the kinetochore itself, (d) mitotic exit, and (e) cytokinesis.

**Figure 7.**
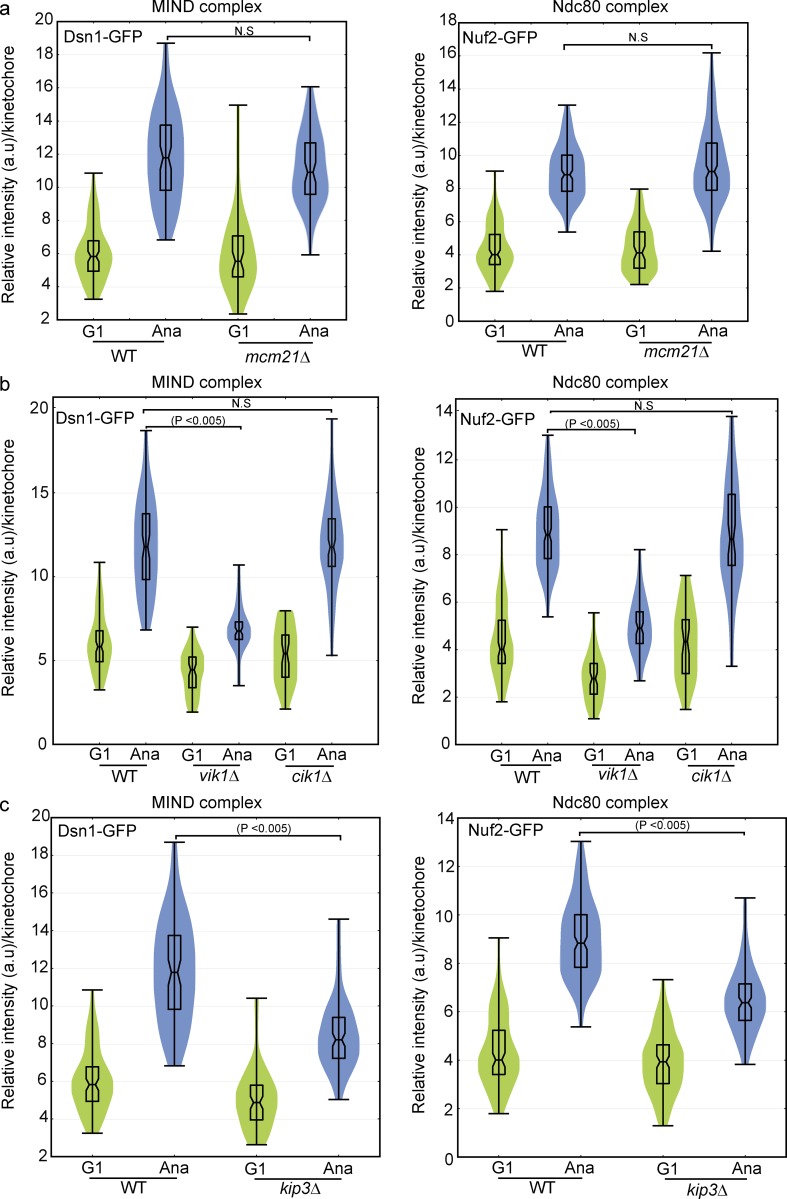
**The kinesin-related motors Kip3 and Vik1 facilitate addition of the MIND and Ndc80 complexes in anaphase.** (a–c) Calibrated imaging was performed for the Dsn1 subunit of MIND and the Nuf2 subunit of Ndc80 in various mutant backgrounds and represented as violin plots. The horizontal middle line is the mean, and the box represents the SD. A two-tailed *t* test was used to test for statistical significance. 80–150 clusters from three independent experiments were used to calculate the copy number. (a) Deletion of *MCM21* did not affect the copy number of Dsn1 or Nuf2 in G1 or anaphase. (b) Disrupting the localization of Kar3 at SPBs with deletion of *VIK1*, but not the midzone localization with deletion of *CIK1*, mildly reduced the copy number of Dsn1 and Nuf2 in G1 and more significantly in anaphase. (c) Deletion of *KIP3* reduced the addition of Dsn1 and Nuf2 in anaphase.

Deletion of *MCM21*, which encodes a subunit of the COMA subcomplex, reduces cohesion at centromeres (Ng et al., 2009; Stephens et al., 2013), which results in lower tension between sister centromeres. We asked whether tension during metaphase was an important factor for structural changes in the kinetochore during anaphase. However, we found that addition of Ndc80 and MIND complexes in anaphase occurred normally without *MCM21* (Fig. 7 a). Mutation in the tension sensor *IPL1* similarly did not affect addition of Ndc80 in anaphase (Fig. S5 a). Experiments with temperature-sensitive mutants that were conducted at 37°C precluded calibrated imaging because of the instability of EGFP, but measurements of intensity from metaphase to anaphase revealed whether the intensity increased during anaphase. Although data are shown for one cell, the results described represent the trend observed in a minimum of eight total cells from three independent experiments. The results of the *mcm21Δ* and *ipl1-2* mutants together suggest neither metaphase tension nor the sensing of the tension are necessary to achieve the anaphase kinetochore configuration.

Kar3 is a kinesin-14 motor protein that depends on noncatalytic motorlike proteins for its localization. Kar3 binds to Vik1 (Mayer et al., 2004) to localize on SPBs and interacts with Cik1 for its midzone localization (Tytell and Sorger, 2006). Kar3-Cik1 can bind to the Ndc80 complex to localize on microtubules (Mieck et al., 2015). Calibrated imaging in deletion mutants revealed that Vik1, but not Cik1, is required for the addition of the MIND subunit Dsn1 and the Ndc80 subunit Nuf2 during anaphase (Fig. 7 b). These results suggest that the SPB function of Kar3-Vik1, but not Kar3-Cik1 at the spindle midzone, can influence structural changes in the kinetochore during anaphase. Although the observations with deletion of *VIK1* could be caused by increased or mislocalized Kar3-Cik1, we did not see evidence of this when monitoring Kar3. Loss of *VIK1* is associated with increased mitotic chromosome loss (Daniel et al., 2006); we speculate this loss could be caused in part by failure to achieve a kinetochore configuration that can effectively track kinetochores.

Kip3 (MCAK in mammals) is a multifunctional motor protein with microtubule-depolymerizing activity for growing microtubules (Su et al., 2011). Kip3 has been implicated in the maintenance of genome stability (Duffy et al., 2016). Calibrated imaging in a *kip3*Δ background revealed that Kip3 was required for the full addition of the MIND subunit Dsn1 and Ndc80 subunit Nuf2 during anaphase because significantly less addition was observed (Fig. 7 c). This finding is consistent with the Stu2 result and suggests that a lower rate of microtubule depolymerization may be sensed and fewer couplers may be added during anaphase. In summary, we found that some motor-related proteins are required for the increase in copy number of MIND and Ndc80 complexes in anaphase. Moreover, there are intermediate levels of addition of these proteins in different mutant backgrounds, reinforcing the idea that the kinetochore structure is adjustable and plastic.

**Figure 8.**
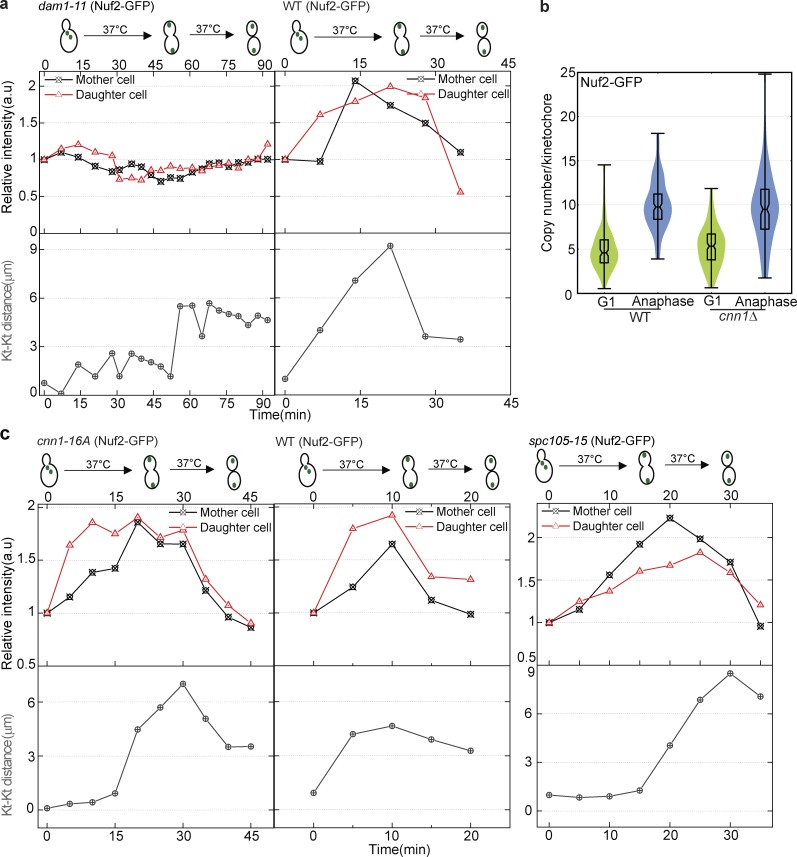
**Dam1 is required for the increase in Nuf2-GFP in anaphase, but the increase occurs normally in *cnn1Δ*, *cnn1-16A*, and *spc105-15* mutants.** (a–c) α-Factor–arrested cells were released at 37°C for live-cell microscopy and were imaged from metaphase. At least eight cells total were imaged from three independent experiments. The Nuf2-GFP subunit of the Ndc80 complex was followed from metaphase to anaphase. (a) In the *dam1-11* temperature-sensitive mutant background at a nonpermissive temperature (37°C), Nuf2-GFP was not added during anaphase. (b) Calibrated imaging was used to calculate and compare the copy number for Nuf2 in WT and *cnn1Δ* strains in G1 and anaphase. Deletion of *CNN1* did not affect the copy number of Nuf2 in either G1 or anaphase. (c) A mutant version of Cnn1 that cannot be phosphorylated (Cnn1-16A) strongly interacted with the Ndc80 complex (Malvezzi et al., 2013). Quantification of intensity of Nuf2-GFP in the *cnn1-16A* mutant background shows addition in anaphase. Quantification of intensity of Nuf2-GFP in the *spc105-15* temperature-sensitive mutant background shows that Nuf2-GFP is added in anaphase. Kt, kinetochore.

The kinetochore structure has been proposed to be hierarchical, with the recruitment of some subcomplexes dependent on the recruitment of others (Biggins, 2013). Deletion of the Mcm21 subunit of COMA did not affect the addition of MIND or Ndc80 in anaphase. We further analyzed whether the addition of MIND and Ndc80 subunits in anaphase was dependent on the microtubule-interacting proteins Dam1 or Spc105. Interestingly, in the *dam1-11* strain, the Nuf2 subunit of Ndc80 did not show addition in anaphase, suggesting that Dam1 complex is necessary for the addition of the Ndc80 complex in anaphase (Fig. 8 a). In the *spc105-15* strain, the Nuf2 subunit of Ndc80 displayed a similar intensity increase in anaphase as in a WT strain (Fig. 8 c).

Mitotic exit requires the function of the protein phosphatase Cdc14 (Jaspersen et al., 1998; Visintin et al., 1998). The *cdc14-3* mutant arrests in late anaphase (Culotti and Hartwell, 1971). In the *cdc14-3* strain, the Nuf2 subunit of Ndc80 increased similar to a WT strain (Fig. S5 b). The strain then arrested with the anaphase configuration, demonstrating that the return to the minimal configuration of the kinetochore requires progression of the cell cycle into telophase.

Cytokinesis requires components of the septin ring such as Cdc10 and Cdc11 (Hartwell, 1971; Longtine et al., 1996; Field and Kellogg, 1999). Without septin function, cytokinesis does not occur, but other aspects of the cell cycle may still progress. In *cdc11-4* and *cdc10-5* strains, which did not undergo cytokinesis, the Nuf2 subunit of Ndc80 increased similar to a WT strain and then returned to the minimal configuration of the kinetochore, again similar to a WT strain (Fig. S5 c). This result suggests that the physical act of cytokinesis is not required for the structural transitions in the kinetochore. Collectively, these results demonstrate that the structural transitions at the kinetochore are coupled to some of the previously studied cell cycle regulators, but not to others.

### Kinetochore intensity increase during anaphase is evolutionarily conserved

**Figure 9.**
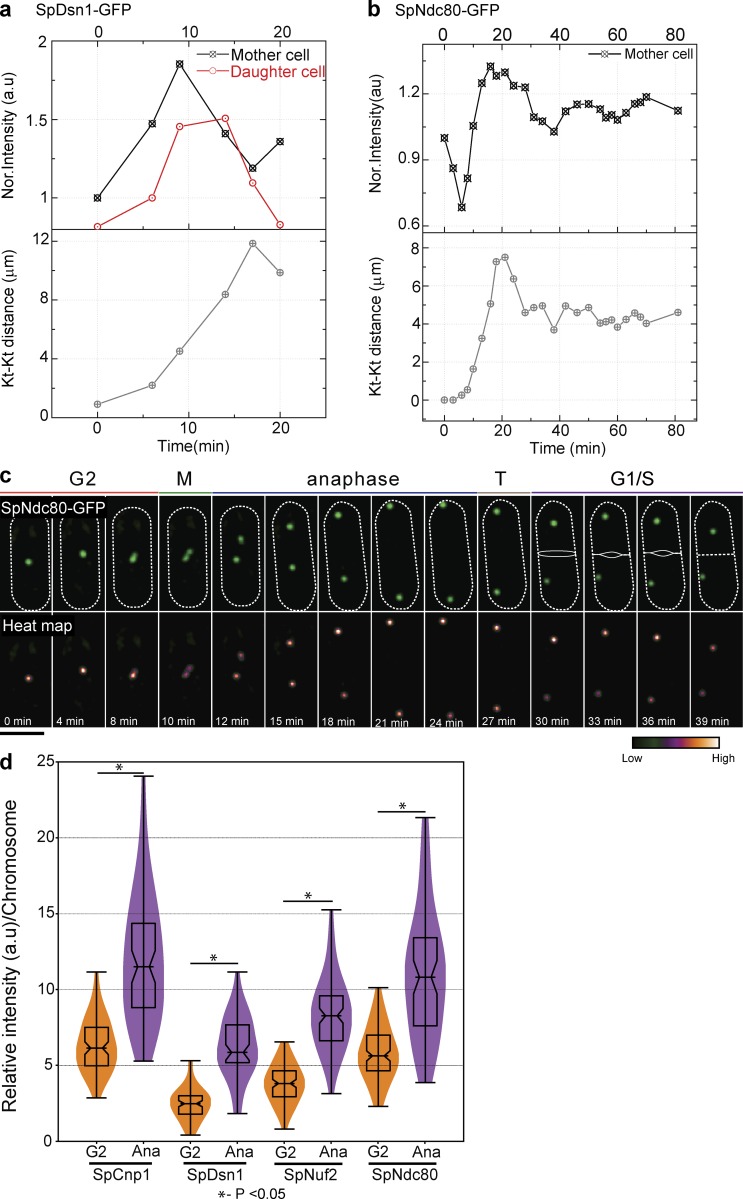
**Ndc80 increase during anaphase is observed in *S. pombe*.** (a) Quantification of intensity of SpDsn1-GFP (MIND–MIS12 complex) from metaphase to anaphase from a single cell shows an increase during anaphase (*n* = 8). (b and c) Quantification (b) and snapshots (c) showing an increase in intensity of SpNdc80-GFP of a single cell from metaphase to anaphase in fission yeast (*n* = 8). Bar, 5 µm. (d) Quantification of kinetochore (Kt) protein intensity per chromosome in G2 and anaphase represented as a violin plot. Kinetochore clusters in anaphase have significantly higher intensity than kinetochore clusters in G2. 50–130 clusters were used for the violin plot. A two-tailed *t* test was used to test for statistical significance.

Budding and fission yeast diverged ∼600 million years ago from a common ancestor (Hedges, 2002). Fission yeast has a regional centromere but forms a subdiffraction-limited kinetochore cluster like budding yeast (Liu et al., 2005), and the kinetochore–microtubule architecture is also conserved (Joglekar et al., 2008a). We tagged kinetochore proteins with EGFP to study their intensity during the cell cycle in *Schizosaccharomyces pombe*. Dsn1-EGFP and Ndc80-EGFP intensities increased in anaphase compared with G2 (Fig. 9, a and b), similar to our observations in budding yeast (Figs. 1 and S1). We used the kinetochore distance to identify the cell cycle stages. For technical reasons, it was easier to quantify the intensity in G2 (rather than metaphase) and anaphase and normalize for the number of chromosomes. The intensity of the kinetochore cluster dropped as the cell transitioned from G2 to metaphase and then increased again during anaphase (Fig. 9 c). Anaphase cells have higher fluorescence intensity per chromosome for subunits of the MIND and Ndc80 subcomplexes, suggesting more copies per chromosome in anaphase (Fig. 9, a–d). The Dam1 complex in *S. pombe* localized along the microtubule with multiple puncta (Gao et al., 2010), making it difficult to assess kinetochore-associated Dam1. Altogether, our data suggest that the Ndc80 and MIND subcomplexes also increase during anaphase in fission yeast, suggesting that plasticity of kinetochore structure may be evolutionarily conserved.

### Kinetochore copy number increase is predicted to improve chromosome attachment

**Figure 10.**
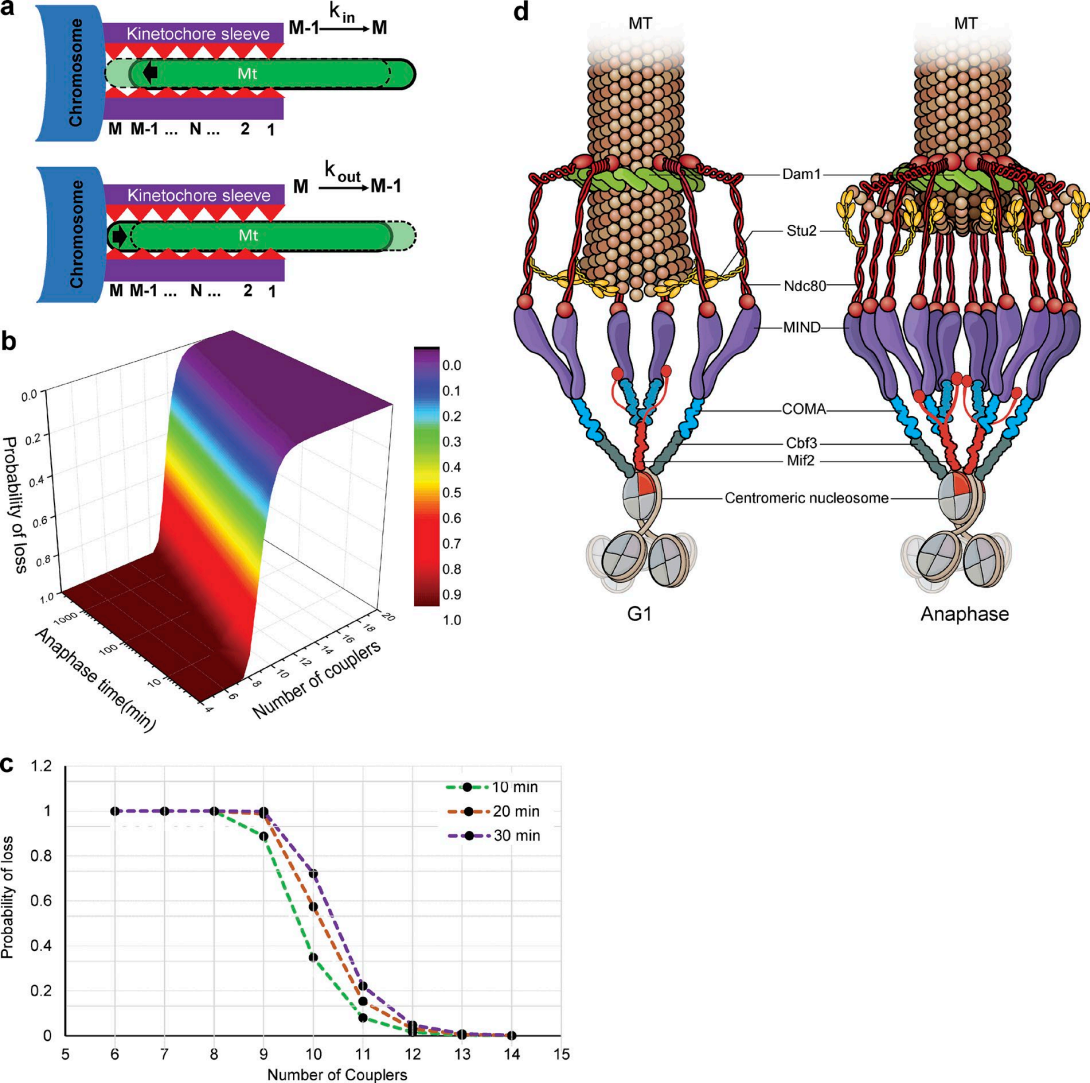
**Simulations using Hill’s biased diffusion model and a working model for kinetochore configuration in G1 and anaphase.** (a) Hill’s equation for the microtubule (MT)–kinetochore interaction is shown with a cartoon to depict each variable. During microtubule polymerization, the microtubule moves inside the kinetochore sleeve from *M* − 1 to *M* for calculating the *k_in_*. During depolymerization of the microtubule, interactions are broken and reformed in a new position by moving from *M* to *M* − 1 for calculating the *k_out_*. (b) The number of couplers is plotted against the probability of detachment (shown by heat map) as a function of anaphase length. Addition of each coupler increases the probability of remaining attached exponentially. (c) The probability of an attachment breaking is plotted against the number of couplers for a 10-, 20-, and 30-min anaphase, revealing that the addition of each coupler exponentially decreases the loss of kinetochore–microtubule interaction. (d) The kinetochore in G1 through metaphase has fewer copies of COMA, MIND, and Ndc80 subcomplexes relative to the kinetochore at the end of anaphase, which experiences addition of the Okp1–COMA (blue), Mtw1–MIND (purple), and Ndc80 (red) subcomplexes. This addition is facilitated by Stu2 (yellow). The Dam1 complex (green) is represented as a similar ringlike structure in G1 and anaphase encircling the microtubule.

In anaphase, the kinetochore must persistently track the rapidly depolymerizing microtubule. Hill’s model for kinetochore–microtubule interaction has been extensively used to mathematically describe kinetochore movement (Hill, 1985). The number of proximal coupler microtubule attachments can change based on microtubule dynamics, with depolymerization favoring motion, and polymerization favoring a stable attachment (Hill, 1985; Joglekar and Hunt, 2002; Joglekar et al., 2006; Asbury et al., 2011). Polymerization of the microtubule changed the kinetochore binding site from N to N + 1, promoted by random thermal motion, whereas depolymerization promoted the movement of the microtubule out of the kinetochore “sleeve” (Fig. 10 a; Joglekar and Hunt, 2002). Given our experimental observations, we simulated kinetochore–microtubule attachment during prolonged depolymerization with different numbers of couplers (a.k.a. different sleeve lengths), with the speculation that more couplers might prevent detachment of the kinetochore from the disassembling microtubule. Our simulation studies using Hill’s equation show that the addition of couplers decreased the probability of detachment exponentially in a given anaphase time (Fig. 10 b). If we simulated a 10-min anaphase, addition of a single coupler decreased the probability of a lost attachment by approximately fourfold. The model predicts that the same number of couplers will have a lower probability of loss with longer anaphase (Fig. 10 c). Based on our experimental evidence, the coupler that could be added to prevent a lost attachment is the Ndc80 submodule. The simulations demonstrate that adding even a single coupler is predicted to have a significant effect on the persistence of attachment.

## Discussion

Error-free chromosome segregation is essential for successful progression of life. Experiments using purified yeast kinetochores have revealed that microtubule depolymerization makes kinetochores highly susceptible to detachment (Akiyoshi et al., 2010), which in turn could be deleterious for chromosome transmission. Our work suggests that the kinetochore structure, particularly the MIND and Ndc80 subcomplexes but not the Dam1 subcomplex, is modified during the rapid microtubule depolymerization of anaphase in living cells. The increase in Ndc80 copies has the potential to increase contacts between the microtubule and kinetochore during anaphase, presumably to facilitate efficient tracking and prevent chromosomes from being lost from the spindle. We speculate that fewer copies of the Ndc80 submodule during G1 and metaphase with the kinetochore in a minimal configuration could facilitate correction of spindle–microtubule misattachments as compared with the anaphase configuration, in which more kinetochore–microtubule contacts would need to be broken before correction (Fig. 10 d). Our results suggest that the kinetochore is an adjustable structure, which may contribute to robust functionality for chromosome segregation.

### Kinetochore submodules are adjustable and stoichiometric during anaphase

The kinetochore interacts with the assembling and disassembling microtubule tip throughout the cell cycle. During anaphase, however, the kinetochore must track on a rapidly disassembling microtubule without losing the attachment. The Ndc80 complex makes this connection to the microtubule tip through its calponin homology domain and N-terminal unstructured fingerlike projections. The Ndc80 complexes align parallel with each other, with the highest Förster resonance energy transfer in anaphase (Aravamudhan et al., 2014). As a microtubule disassembles, it is proposed that the Ndc80 complex breaks the interaction with the microtubule near the tip and forms a new, more internal interaction (Asbury et al., 2011; Foley and Kapoor, 2013). Oligomerization of the Ndc80 complex is necessary for it to track the microtubule in vitro. Dam1, an essential complex for kinetochore function in budding yeast, forms a ringlike structure and tracks the disassembling microtubule. The presence of these two different types of microtubule contacts may facilitate robust attachment. However, the type of attachments made by the Ndc80 submodule may be more amenable to adjustment. We observe that the Ndc80 complex gradually adds copies as the SPBs separate during anaphase, and this addition is accompanied by the addition of copies of the MIND complex and the Okp1 and Ame1 subunits of the COMA complex (Hornung et al., 2014), which anchors the MIND complex to the centromere. Collectively, Okp1/Ame1-MIND-Ndc80 may form a working structural module, stretching from the inner kinetochore to the microtubule. The addition of the Okp1/Ame1-MIND-Ndc80 kinetochore submodules could promote chromosome attachment to microtubules as chromosomes move to opposite poles during anaphase.

The relative stoichiometry of proteins within Ndc80 and MIND submodules remains similar in G1 and anaphase, reinforcing the idea that they function as structural submodules within the kinetochore. Because there are more copies of Ndc80 than MIND and more copies of MIND than Okp1/Ame1, our results would suggest a single Okp1-Ame1 submodule may associate with more than one MIND submodule, and one MIND submodule may associate with more than one Ndc80 submodule. Although Okp1 and Ame1 may exist in the COMA submodule with Ctf19, they have a significantly higher copy number than Ctf19. However, overall stoichiometry of the COMA submodule is roughly maintained from G1 to anaphase. In contrast to the subunits of the MIND and Ndc80 submodules, which all exhibit similar behavior in FRAP and photoconversion experiments, Okp1 and Ame1 show more recovery than Ctf19 after photobleaching, suggesting different structural dynamics for the subunits of this submodule. Cnn1 has been reported to function as a receptor for the Ndc80 complex in anaphase (Schleiffer et al., 2012; Malvezzi et al., 2013). However, neither deletion of *CNN1* nor the phosphodeficient mutation *cnn1-16A* affected the copy number or addition of Ndc80, suggesting that it is not required for recruitment of Ndc80 in anaphase (Fig. 8, b and c). Although Cnn1 may bind to Ndc80 and normally be involved in its recruitment during anaphase, it does not appear to be essential. Overall, our results start to reveal the behavior of subunits and submodules within the structure of the living kinetochore.

The Dam1 subcomplex forms an oligomeric structure and at high protein concentrations, it forms a complete ring with 16-fold symmetry in vitro (Westermann et al., 2005; Miranda et al., 2007; Wang et al., 2007; Ramey et al., 2011). The Dam1 subcomplex can interact with the microtubule as a monomer, partial ring, complete ring, or double ring in vitro (Miranda et al., 2007; Gestaut et al., 2008; Kim et al., 2017), suggesting that multiple modalities of interaction are possible. The recently proposed model of two Dam1 rings, based on in vitro data, is difficult to reconcile with our observations along with those of Joglekar et al. (2006), which were collected in vivo. The data from Kim et al. (2017) could instead be interpreted to mean that the Dam1 complex can interact with microtubules in two distinct modes, rather than two Dam1 rings coexisting in vivo. All forms of the Dam1 subcomplex form a load-bearing contact with the microtubule and track the depolymerizing microtubule processively (Gestaut et al., 2008; Umbreit et al., 2014). Previous imaging estimated the Dam1 subcomplex at 10–20 copies per kinetochore in vivo (Joglekar et al., 2006), similar to our estimate of 12 ± 4 copies. A partial Dam1 ring has been observed by tomography (McIntosh et al., 2013), suggesting that the Dam1 complex could form an incomplete ring in vivo. Our data support the idea that Dam1 may be an incomplete ring in vivo and, in contrast with Cnn1, that Dam1 is required for the addition of Ndc80 in anaphase.

Previous studies on kinetochore protein copy number specifically focused on metaphase or anaphase and typically used centromeric histone protein Cse4 as a reference (Joglekar et al., 2006, 2008a). The relative copy number of kinetochore proteins was given in metaphase and anaphase relative to Cse4 in metaphase and Cse4 in anaphase, respectively, with Cse4 unchanged over the cell cycle. However, a later study argued that Cse4 fluorescence changes between metaphase and anaphase (Shivaraju et al., 2012), suggesting these previous measurements may be reinterpreted. With the unchanging Cse4 reference used in the previous study, the anaphase values calculated relative to metaphase showed a slight relative decrease in copy number for the MIND complex member Mtw1p, a slight relative decrease for COMA complex member Ctf19p, and a large (approximate factor of 2) relative decrease in copy number for Dam1 complex member Ask1p (Joglekar et al., 2006). Based on our collection of calibrating imaging, FRAP, and photoconversion data, we instead argue that subunits of the Dam1 subcomplex remain constant from metaphase to anaphase, whereas subunits of the MIND and COMA submodules are added during anaphase. Our data are therefore consistent with the previous study but simply differ in the calibration reference. In fact, if we use Cse4 as a reference at two copies/kinetochore in anaphase, our anaphase numbers are strikingly similar to the previous study (Table S2). Our FRAP results are also consistent with prior studies. Previous FRAP experiments were performed on a handful of kinetochore proteins for ∼5 min (300 s) and did not reveal any recovery (Joglekar et al., 2006, 2008b; Suzuki et al., 2016). However, these experiments differed from ours in that they were performed either in metaphase or in anaphase. In contrast, we followed recovery from metaphase to anaphase typically for a longer period. Furthermore, the critique that compaction of the cluster can affect the intensity of the spot by 40% and therefore may explain the differences we observe in anaphase is refuted by both the FRAP and photoconversion data, where intensity changes are not the readout but rather are the absence (Dam1 complex) or presence (Ndc80 complex, for example) of intensity in anaphase after photobleaching or photoconversion in metaphase. In sum, our results are compatible with the published literature.

### Kinetochore plasticity may be influenced by microtubule dynamics and may be evolutionarily conserved

MAPs can influence the rate of microtubule assembly and disassembly during the cell cycle (Kosco et al., 2001). The Stu2/XMAP215 family is important for spindle elongation and kinetochore–microtubule interaction (Usui et al., 2003; Miller et al., 2016). Loss of Stu2 severely reduces the microtubule dynamics, makes spindles shorter, and arrests cells in metaphase (Kosco et al., 2001; Pearson et al., 2003). Reduced microtubule dynamics in a *stu2* mutant (McAinsh et al., 2003) as well as the *kip3* mutant (Tytell and Sorger, 2006; Gardner et al., 2011) are associated with less addition of Ndc80 subunits in our experiments, revealing a correlation between a lower disassembly rate and the number of Ndc80 submodules added. Stu2 may help to directly recruit or stabilize additional Ndc80 complexes via its interaction with Ndc80. Given that Stu2 is evolutionarily conserved (human orthologue ch-TOG) and that its interaction with Ndc80 is evolutionarily conserved (Miller et al., 2016), this protein could play a similar role in higher eukaryotes. In contrast, the Dam1 complex was unaffected by compromising microtubule dynamics. We speculate that the kinetochore may be able to sense the rate of microtubule depolymerization and adjust the number of Ndc80 copies added for attachment, whereas the Dam1 subcomplex may not vary its attachment by depolymerization rate. The kinetochore configuration in the *stu2-11* and *kip3* mutants during anaphase is intermediate between the normal G1 and anaphase configurations, suggesting fewer microtubule couplers are present under conditions of reduced depolymerization speed and reinforcing the idea of structural plasticity.

A purified kinetochore complex detaches from a disassembling microtubule ∼100-fold faster than a polymerizing microtubule in vitro (Akiyoshi et al., 2010; Sarangapani et al., 2014); detachment in a cell could result in aneuploidy. Our results suggest that the living kinetochore enhances its interaction with the depolymerizing microtubule by increasing the copy number of the Ndc80 subcomplex. These structural changes would be difficult to observe in vitro because they depend on a soluble pool of subunits. Simulation of kinetochore interactions with microtubules predicts that the addition of each coupler decreases the probability of losing the microtubule attachment by fourfold. We propose that the addition of the Ndc80-MIND-Okp1-Ame1 modules could serve this role. In yeast, as each chromosome is attached to a single microtubule, this addition may be especially important for faithful completion of chromosome segregation. Our results in fission yeast support addition of Ndc80 subcomplexes during anaphase as a conserved feature of a living kinetochore structure.

### How might structural plasticity promote chromosome segregation?

The structural plasticity of the kinetochore may contribute to its functionality. We speculate that in metaphase, fewer copies of Ndc80 might facilitate error correction by Aurora B kinase (Biggins et al., 1999; Cheeseman et al., 2002; Tanaka et al., 2002). Increased Ndc80 levels, as reported in cancer, may compromise error correction if they lead to increased microtubule attachments, contributing to the increased aneuploidy observed (Yuen et al., 2005; Pfau and Amon, 2012; Meng et al., 2015). Once chromosomes start moving to the poles during anaphase, the addition of Ndc80-MIND-Okp1-Ame1 submodules may provide extra attachments that facilitate robust microtubule tracking. Interestingly, kinetochores revert to the lower copy structure in telophase/G1. Many kinases and phosphatases regulate mitosis and the kinetochore in particular. Our examination of metaphase and anaphase regulators revealed that neither intersister tension nor the sensing of tension are required for the structural changes, but factors that affect microtubule dynamics, the kinetochore itself, and mitotic exit promote the structural transitions. We speculate that additional kinases and/or phosphatases will contribute to the regulation of the kinetochore configuration. In the future, it will be important to pursue how the living kinetochore structure is regulated and how the structural plasticity contributes to its functionality for chromosome segregation.

## Materials and methods

### Yeast strains

The *S. cerevisiae* and *S. pombe* strains used in this study are listed in Table S1. We thank S. Westermann (University of Duisburg-Essen, Essen, Germany) for providing yeast strains. Karyotyping by quantitative PCR was done as previously described (Pavelka et al., 2010) to examine the ploidy level of strains used in microscopy studies. In brief, genomic DNA from experimental and control haploid and diploid strains was used to perform a quantitative PCR assay with a set of primers from one noncoding region on each chromosome arm to determine the copy number of the PCR product in the experimental strain. Reaction conditions, primers, and chromosome copy number calculations were previously derived (Pavelka et al., 2010).

### Microscopic techniques

All microscope data for GFP counting, FCS, and FRAP were acquired as previously described (Shivaraju et al., 2012) by using a LSM-510 confocal microscope (ZEISS) outfitted with a ConfoCor 3 module and two single-photon–counting avalanche photodiodes. A C-Apochromat 40× 1.2 NA water objective was used. A high-frequency trading 488/561 main dichroic allowed excitation of GFP (488-nm laser line) and mCherry (561-nm laser). A secondary nearfield transducer 565 beam splitter was used as an emission dichroic. After passage through a 505–550-nm Bandpass or Longpass 580 filter for GFP and mCherry, respectively, photon counts were collected on avalanche photodiodes in single-photon counting mode. Pinhole was set to 1 airy unit.

### FCS with image calibration

FCS and image calibration were done as previously described (Shivaraju et al., 2012). In brief, using the FCS module of the ConfoCor 3 (ZEISS), FCS of an endogenously expressed monomeric GFP in live cells was used to calculate the intensity of a single GFP. As with imaging, the 488-nm laser line was used to excite GFP, and emission was collected through a 505–550-nm Bandpass filter. Pinhole was set to 1 airy unit. To calculate the copy number of a kinetochore protein, a z series was taken with 0.4-µM step size and 6.4-µs pixel dwell time. The kinetochore cluster was fit to a 2D Gaussian, and peak amplitude of the fit was divided by single GFP intensity after correcting for differences in laser power. Fits were performed with custom ImageJ (National Institutes of Health) plugins incorporating a grid search over spatial and width coordinates and linear least squares for amplitude and baseline determination. Automation was accomplished with ImageJ macros included in the online supplemental material. To compare values using the integrated intensity (Joglekar et al., 2006), we multiplied the spot intensity amplitude by the SD squared of the Gaussian fit and then normalized to a value of 2 for Cse4 intensity in anaphase. Although the integrated intensity method compared with a known standard and the amplitude-based method that determines GFP copy number based on comparison to FCS of a cytosolic GFP standard should provide identical results, we prefer the amplitude-based method for two reasons. The first reason is that it calculates the copy number from first principles and does not require a standard for comparison. The second reason is that when fitting noisy data to a 2D Gaussian, there is far less uncertainty in the height of the Gaussian (amplitude) than there is in the width of the Gaussian (SD). Furthermore, the integral of a Gaussian uses the SD squared, adding more uncertainty. The main drawback to the amplitude-based method is that it requires the spot being fit to be diffraction limited. This drawback is partially mitigated in this work by use of a 1.2-NA objective rather than a 1.4-NA objective, effectively increasing the size of a spot that can be considered diffraction limited (Smith et al., 2014) as well as the removal of spots above a certain size threshold as described previously (Shivaraju et al., 2012). A Python script with MatPlotLib was used to plot the kinetochore copy number as a violin plot. Origin 9.1 was used for statistical analysis.

### FRAP

FRAP measurements were performed to examine the recovery of kinetochore protein during anaphase. Yeast cells expressing kinetochore proteins tagged with EGFP were grown to mid-log phase in synthetic complete media, harvested, and sandwiched between a slide and coverslip in a 1% agarose solution made with medium. Long time-lapse imaging demonstrated yeast cells were alive and divided at a normal rate in the agar pad for up to 4 h. We took time points with 5-min intervals to minimize bleaching. Before photobleaching, a z series was taken with 0.4-µM step size and 6.4-µs pixel dwell time. Acquisition of a z stack was essential because of the mobility of the kinetochore cluster in living yeast cells and to ensure proper quantitation of kinetochore intensity. After the initial acquisition, a kinetochore cluster labeled with EGFP was irreversibly photobleached by four rapid scans with high 488-nm laser power. The ability of the cells to continue to grow and divide ensured that photobleaching did not grossly damage the cells. After photobleaching, videos were acquired to examine recovery of the kinetochore cluster during anaphase. In most cases, cells were used that also expressed Spc42-mCherry from a centromeric plasmid to mark the cell cycle. Recovery of kinetochore proteins was observed as the reappearance of a punctate spot centered in the nucleus. ImageJ software was used as in the previous section for calibrated imaging to calculate the intensity, and the distance between the kinetochore clusters was determined with standard ImageJ measurement tools. The percent recovery was calculated as follows:

$$\begin{array}{l} \%Recovery=\\ \left(\frac{Max.\;Intensity\;in\;anaphase-Post_{bleach}\;Intensity}{Pre_{bleach}\;Intensity-Post_{bleach}\;Intensity}\right)\times 100. \end{array}$$

### Photoconversion of kinetochore proteins at metaphase

For cell cycle time series and photoconversion studies, an Ultraview VoX spinning-disk system (PerkinElmer) with a CSU-10 spinning-disk (Yokogawa Electric Corporation) was used. The system was attached to a 200-m inverted microscope (ZEISS). Images were acquired with a 100× 1.46 NA α-Plan Apochromat oil objective onto an electron-multiplying charge-coupled device camera (C9100-13; Hamamatsu Photonics) using Volocity software (PerkinElmer). GFP and mCherry or the green and red forms of tdEos were excited with the 488-nm and 561-nm laser lines, respectively, using a 405/488/561/640 dichroic. The emission filter for green was a 500–550-nm Bandpass, and for red it was a 415–475-nm/580–650-nm dual Bandpass filter. Data were acquired with alternative excitation and were verified to be free of spectral cross-talk.

Photoconversion experiments were used to measure the addition of new protein at the kinetochore cluster in anaphase. Yeast cells expressing tdEos or mEos were grown, and the experiment proceeded as in FRAP—with the exception that the kinetochore cluster was photoconverted with four iterations of the 405-nm laser and subsequently imaged with the 488-nm (green)/561-nm (red) laser line with spinning-disk microscopy using the system detailed above. Intensity quantification with Gaussian fitting was performed as in the calibrated imaging experiments. Percent green addition was calculated as follows:

$$\begin{array}{l} \%Green\;addition=\\ \left(\frac{Max.\;Green\;Intensity\;in\;anaphase-Post_{activation}\;Green\;Intensity}{Pre_{activation}\;Green\;Intensity-Post_{activation}\;Green\;Intensity}\right)\times 100. \end{array}$$

For cell-cycle series/videos, the cells were maintained on 2% agar pads at room temperature.

### Simulation of kinetochore–microtubule interaction with Hill’s equation

Simulations of kinetochore–microtubule tracking for depolymerizing microtubules were performed according to the Hill model (Hill, 1985) as described previously by Joglekar and Hunt (2002) with minor modifications outlined in Fig. 10 a. The microtubule movement inside the sleeve was performed by discrete steps of the size *L* = 0.615 nm, and the size of the sleeve was *M* × *L*. The position *N* of microtubule is determined by its left tip 1 ≤ *N* ≤ *M* (Fig. 6 a).

The following physical mechanisms are incorporated into the model: (a) Random thermal motion of the sleeve at a rate κ. (b) Loss of tubulin monomers at the microtubule tip. Each additional interaction of the microtubule with the tubulin binding site (corresponding with the increasing *N*) reduces the free energy by *W* (it has negative value) so that the insertion of the microtubule into the sleeve is promoted. This movement requires first to break all existing binding between microtubules and binding sites, which creates a potential energy barrier *B* for each occupied binding site so that this barrier is equal to *N* × *B*. We set free energy equal to zero when the microtubule detaches from the sleeve. Thus, at position *N* = 1, we have it equal to *W* < 0; at *N* = 2, it is *2W*; and at arbitrary *N*, this value is *N* * *W*. Consider first the transition from *N* to (*N* − 1) corresponding with outward movement. It can happen by both reasons mentioned above. The rate of transition from position *N* − 1 to position *N* (out of the kinetochore sleeve composed of *M* binding sites or couplers) is described by the following equation (microtubule depolymerization):

$$\;k_{out}=S\left[\frac{\kappa r^{M-N}}{{ƒ}_{\left(load\right)}}+\;\beta \right].$$

In this equation, κ is the constant describing the sleeve thermal motion between tubulin binding sites, *s* is the exponent incorporating the loss of free energy because of movement out of the sleeve, *r* is the potential barrier associated with bond breaking in the sleeve, *β* is the rate of microtubule depolymerization, and *f* is the Boltzmann load factor coupling the tension force (*F*) on the chromosome to the distance traveled, *l*, as follows:

f=e−F×l2kT.

The first contribution to the rate $k_{out}$ reads

$$\;\kappa \times e^{\left[\frac{W}{k\times T}\right]}\times e^{\left[\frac{-\left(M-N\right)\times B}{k\times T}\right]}/f,$$

where *k* is the Boltzmann constant, *T* = 300*K* is the temperature, and *f* is the Boltzmann factor representing the effect of the load on the kinetics.

$$f=e^{\frac{-F\times L}{2kB\times T}},$$

The numerator represents the mechanical work performed in shifting the sleeve against the tension *F*. We used *f* < 1 corresponding with the case when the tension pulls the microtubule out of the sleeve. The second contribution caused by tubulin loss is estimated as *β* × *e*^[^*^W^*^/(^*^k^* ^×^ *^T^*^)]^, where β is the rate of tubulin depolymerization.

Likewise, the transition from position *N* to *N* − 1 is described as follows (microtubule polymerization):

 kin=κ rM−N ƒS.

The reverse transition from *N* − 1 to *N* corresponding with inward movement is caused by the sleeve motion only and its rate,

$$\;k_{in}=\kappa \times \left(e^{\left[-W/\left(k\times T\right)\right]}\right)\times \left(e^{\frac{-\left(M-N\right)\times B}{\left(k\times T\right)}}\right)\times f.$$

Now, we introduce the quantities$\;\;s=e^{\left[\frac{W}{k\times T}\right]}<1\;$and

$$r=e^{\left[\frac{\left(-B\right)}{k\times T}\right]}<1.$$

Using them, we found *k_out_* = κ * *s* * *r^M^* ^−^ *^N^*/*f* + *β* * *s* and *k_in_* = κ * r^M − N^* *f*/*s*. Note that the factor of s in the denominator of the *k_in_* term is different from previous derivations. This does not change the overall behavior of the system except to scale the tension required for tracking upwards by a factor of ∼5.

We used a tension value of 20 pN for all simulations reported in this study. We used the recommended values from Joglekar and Hunt (2002) for all other parameters except in the case of the number of couplers, *M*, which we varied to observe the changes in dynamics of kinetochore loss. Simulations were coded as custom ImageJ plugins and an automation macro, included in the online supplement. Simulations of kinetochore tracking were initiated with the microtubule fully inserted in the kinetochore sleeve (*N* = M). Simulations were run with a time step of 10 µs and for a maximum length of 10,000 s. At each time step, a random number was generated between 0 and 1. If that random number was <*k_in_* (scaled to the time step increment), the microtubule was inserted one binding site further into the sleeve, with a maximum insertion of *M* binding sites. Otherwise, if the random number was > 1 − *k_out_*, the microtubule was pulled out of the sleeve by one binding site. If at any time this resulted in *n* = 0, the kinetochore was considered lost by the microtubule. 1,000 simulations were performed, and the mean loss time was found for each set of coupler number. The time required to lose the kinetochore was exponentially distributed for all parameter sets in this study. Therefore, the probability of loss within a particular anaphase could be estimated by integrating the exponential distribution up to the anaphase length time:

Ploss=1−e−τlossτanaphase.

We mapped the mean kinetochore loss time as a function of the number of couplers of up to nine couplers. Above that value, the simulations became prohibitively long, but the trend of mean loss time was perfectly exponential, following the equation τ*_loss_* = *e*^−13.1789 + 1.6331 ×^ *^M^*. We then used this formula to extrapolate loss times (and probabilities) for higher numbers of couplers.

### Online supplemental material

Fig. S1 shows a gradual increase of subunits from the MIND and Ndc80 subcomplexes from metaphase to anaphase. Similar intensity measurements for a subunit of the Dam1 subcomplex do not show an increase in intensity during the same cell cycle window. Fig. S2 presents the results of an alternative calculation method for the copy number of kinetochore proteins. Fig. S3 shows quantification of FRAP performed on kinetochore subunits. COMA, MIND, and Ndc80 complex subunits recovered in anaphase, whereas subunits of the Dam1 subcomplex did not. Fig. S4 presents quantification from photoconversion experiments of kinetochore proteins. Fig. S5 presents quantification of Ndc80 addition during anaphase and reduction in G1 in spindle assembly checkpoint, mitotic exit pathway, and cytokinesis mutants. Table S1 is a list of strains used in this study. Table S2 is a comparison of kinetochore copy in anaphase. ImageJ macros and plugins are available in the online supplemental material. The macro entitled “batch_gaussian_roi_macro_v2.ijm” cycles through the point regions of interest in the RoiManager and fits them in the selected channel and at their brightest z position to 2D Gaussians via a grid search and linear least squares methodology. The macro entitled “kt_loss_sim_macro.ijm” runs 1,000 kinetochore-tracking simulations until the kinetochore is lost or the simtime value is met. The macro then outputs a plot of loss times. There are three plugin files (Jay_plugins, Jay_plugins2, and Jay_Plugins3) that provide support code for the macros.

## Supplementary Material

Supplemental Materials (PDF)

File S1 (ZIP)
