# Supplementary material for: Structural plasticity of the living kinetochore
Source: J Cell Biol. 2017 Nov 6;216(11):3551–70. doi: 10.1083/jcb.201703152 (PMC5674893; doi:10.1083/jcb.201703152)
Supplement: Supplemental Materials (PDF) [file JCB_201703152_sm.pdf]

Dhatchinamoorthy et al., https://doi.org/10.1083/jcb.201703152

THE JOURNAL OF CELL BIOLOGY

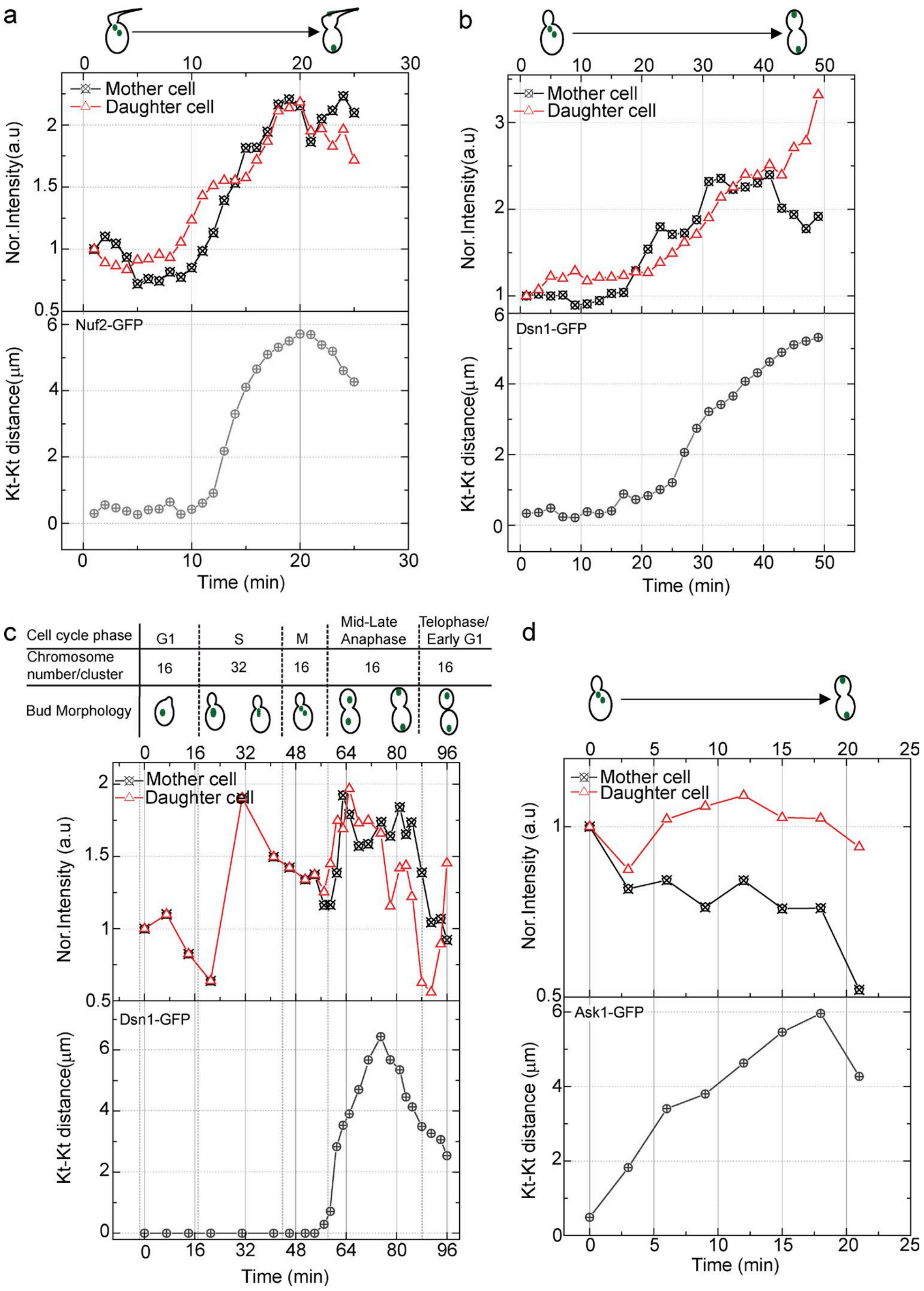

Figure S1. **Subunits in the MIND and Ndc80 subcomplex increase in intensity during anaphase, whereas the intensity of a subunit in the Dam1 subcomplex does not.** (a and b) Intensity of Nuf2-GFP (a) and Dsn1-GFP (b) were followed from metaphase (kinetochore [Kt] cluster distance <0.8 µm) to anaphase/telophase. In this experiment, intensity measurements were collected at very short intervals. The intensity of Nuf2 and Dsn1 increased gradually as the kinetochore clusters separated from each other (n = 10). (c) The intensity of Dsn1-GFP was followed over the cell cycle. α-Factor–arrested cells were released to image the complete cell cycle with more widely spaced time points to prevent photobleaching (n = 8). The intensity of Dsn1 increased during anaphase like the intensity of subunits of the Ndc80 complex. (d) The intensity of Ask1 stayed constant or decreased from metaphase to anaphase.

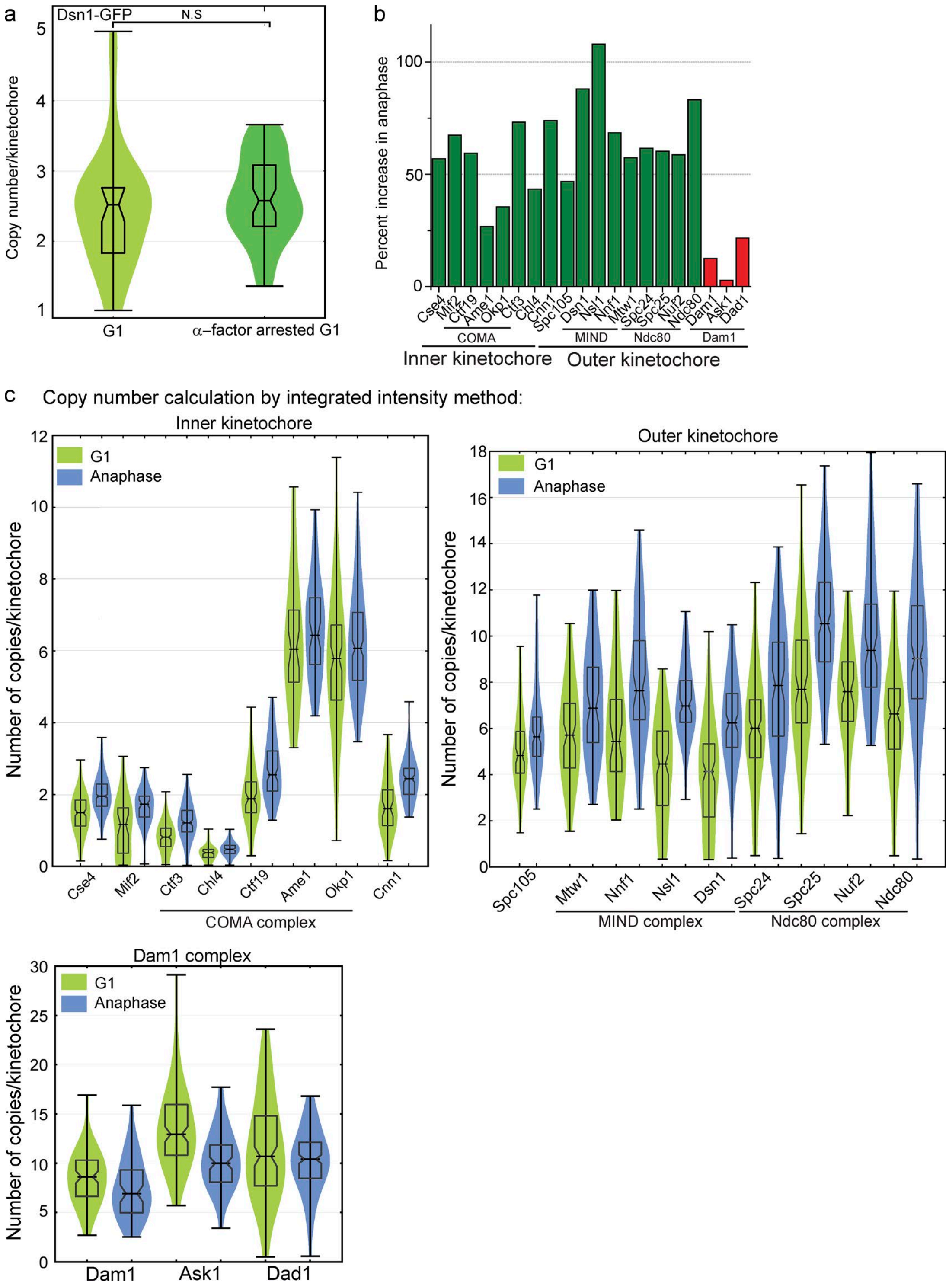

Figure S2. **Copy number of kinetochore proteins in G1 and anaphase calculated by the integrated intensity method.** (a) The copy number of Dsn1-GFP as calculated by calibrated imaging from 100–150 clusters (*n* = 3) was similar for cells in G1 whether derived from an asynchronous culture or α-factor arrest. (b) The percent change in kinetochore proteins from G1 to anaphase is shown (*n* = 3). Subunits of the MIND and Ndc80 subcomplexes typically displayed >50% increase in anaphase. (c) An alternative method for data processing to that shown in Fig. 2, known as integrated intensity (Joglekar et al., 2006), in which Cse4 levels from anaphase were used as a reference to calculate the copy number, also revealed an increase in subunits of the MIND and Ndc80 subcomplexes in anaphase as compared with G1.

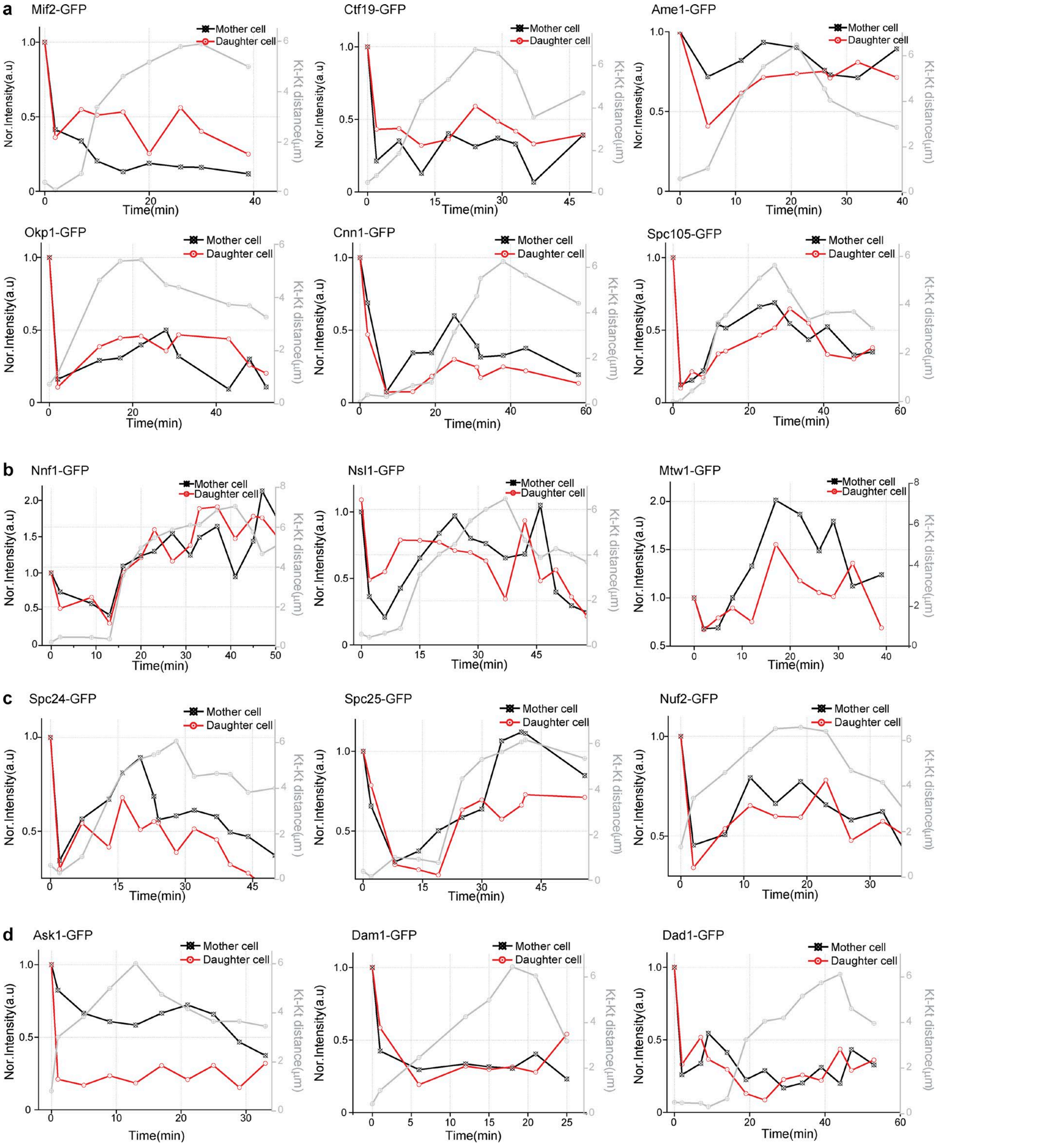

Figure S3. **Quantification of FRAP shows subunits of the COMA, MIND, and Ndc80 subcomplexes recover in anaphase, but subunits of the Dam1 subcomplex do not.** (a) FRAP results are shown for centromere-proximal kinetochore (Kt) proteins Mif2, Ctf19, Okp1, and Ame1. Metaphase cells were used to bleach the kinetochore cluster. Bleached kinetochore clusters were followed through anaphase. Intensity was normalized to 1 at 0 min. The second time point represents the photobleach moment, and each plot represents a single cell from multiple experiments. Mif2 (*n* = 10) and Ctf19 (*n* = 20) did not recover in anaphase, suggesting some of the centromere-proximal kinetochore proteins formed a structure that did not turn over. However, essential proteins in the COMA subcomplex that anchor the MIND subcomplex did recover in anaphase (Ame1 [*n* = 4] and Okp1 [*n* = 14]). Cnn1 (*n* = 25) had some recovery. Spc105 (*n* = 16), which can interact with microtubules, showed recovery in anaphase after bleaching. (b) The MIND subcomplex subunits (Nnf1 [*n* = 11], Nsl1 [*n* = 28], and Mtw1 [*n* = 14]) were subjected to similar FRAP experiments. These displayed >80% recovery in anaphase. (c) Proteins from the Ndc80 subcomplex (Spc24 [*n* = 10], Spc25 [*n* = 25], and Nuf2 [*n* = 9]) recovered in photobleaching experiments in anaphase. Similar recovery of MIND and Ndc80 subunits suggest they were both added as chromosomes moved toward the poles in anaphase. (d) Proteins from the Dam1 subcomplex (Ask1 [*n* = 19], Dam1 [*n* = 17], and Dad1 [*n* = 10]) did not recover in anaphase from photobleaching during metaphase. Anaphase was determined by the distance between the kinetochore clusters.

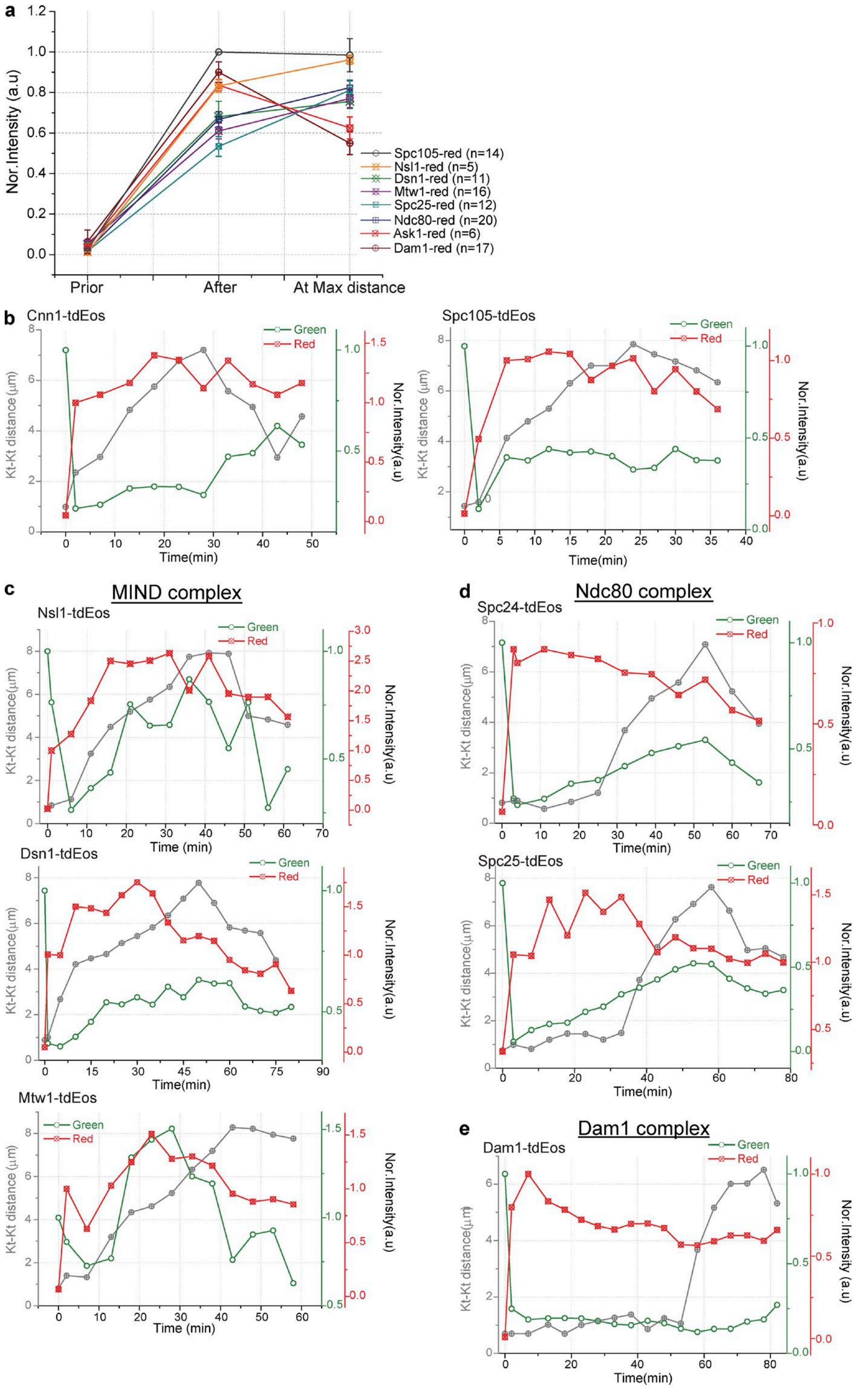

Figure S4. **Quantification of photoconversion experiments for outer kinetochore proteins.** (a) Summary graph showing the retention of red fluorescence at the kinetochore (Kt) in photoconversion experiments, with numbers of experiments indicated in parentheses. Most of the proteins retained a similar level of red fluorescence at anaphase, suggesting the kinetochore keeps its old copies. Dam1 and Ask1 subunits showed a slight loss of red fluorescence, suggesting either a few copies were lost or that there was some minor photobleaching. (b) In metaphase, a kinetochore cluster was photoconverted from green to red and followed through anaphase. Quantification of Cnn1-tdEos and Spc105-tdEos shows addition of new copies in anaphase. (c) Quantification of Nsl1-tdEos, Dsn1-tdEos, and Mtw1-tdEos after photoconversion. Proteins from the MIND subcomplex show addition of a new protein in anaphase. (d) Photoconversion of Ndc80 subcomplex subunits (Spc24 and Spc25) in metaphase revealed addition of copies (green) in anaphase. (e) Photoconversion of Dam1 showed no increase in green fluorescence, indicating that no new protein was added during anaphase. Percent green addition correlated well with the percent FRAP recovery.

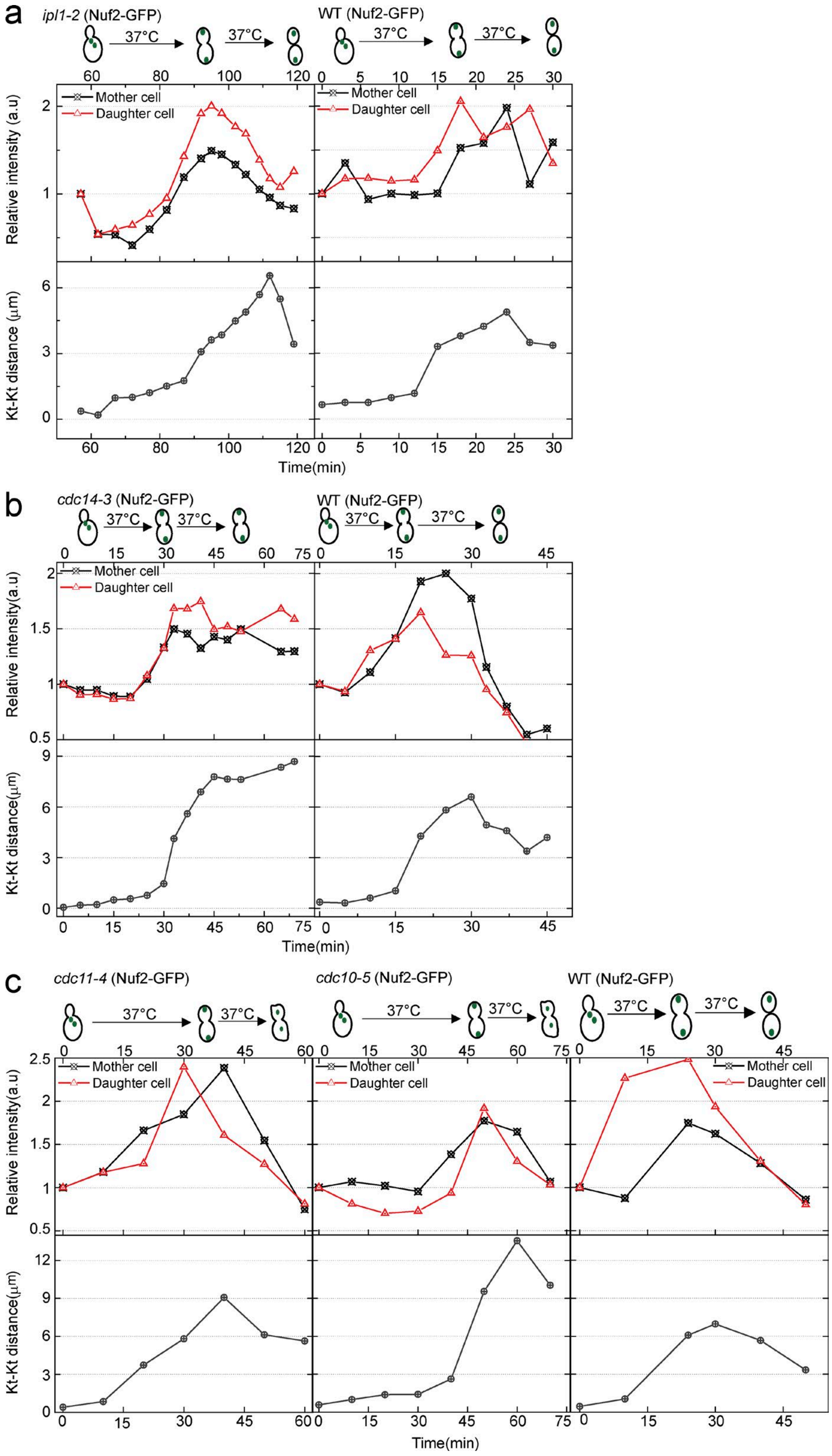

Figure S5. **Tension sensing and cytokinesis are not required for Ndc80 transitions, whereas Cdc14 is required for the transition back to the G1 copy number for Ndc80.** (a–c) α-Factor–arrested cells were released in 37°C for live-cell microscopy and were imaged from metaphase. At least eight cells total were imaged from three independent experiments. (a) Ipl1/Aurora B is not necessary for addition of the Nuf2 subunit of Ndc80 in anaphase. Quantification of the intensity of Nuf2-GFP in the temperature-sensitive mutant *ipl1-2* shows increase in intensity from metaphase to anaphase, similar to WT. (b) Quantification of the intensity of Nuf2-GFP in the *cdc14-3* background at 37°C starting at metaphase shows cells arrest with a high copy number of Nuf2-GFP for ~45 min. (c) Similar quantification of Nuf2-GFP in mutants (*cdc10-5* and *cdc11-5*), which lack cytokinesis at a higher temperature (37°C), shows both the increase in Nuf2-GFP as clusters separate during anaphase and a return to lower intensity as kinetochore (Kt) distance diminishes.

Table S1. **Strains used in this study**

Figure	Strain name	Genotype
**Budding yeast (*S. cerevisiae*)**		
1 b, 2 b, 2 d, 3 e, s2 c	KDH363	*MATa his3Δ1 leu2Δ0 met15Δ0 ura3Δ0 Ndc80-GFP::URA3*
1 c, 2 c, 2 d, 3 e, 6 b, s2 c, s3 d	KDH362	*MATa his3Δ1 leu2Δ0 met15Δ0 ura3Δ0 Dm1-GFP::URA3*
1 d, 1 f, 2 b, 3 a, 6 a, s1 a, s2 c	KDH180.1	*MATa his3Δ1 leu2Δ0 met15Δ0 ura3Δ0 Nuf2-GFP::URA3*
1 e, 1 g, 3 c, 3 d, s1 d, s2 c	KDH233	*MATa his3Δ1 leu2Δ0 met15Δ0 ura3Δ0 Ask1-GFP::HIS3*
2 a, 2 d, s2 c	KDH181.1	*MATa his3Δ1 leu2Δ0 met15Δ0 ura3Δ0 Cse4-GFP::URA3*
2 a, 2 d, 3 e, s2 c, s3 a	KDH364	*MATa his3Δ1 leu2Δ0 met15Δ0 ura3Δ0 Mif2-GFP::HIS3*
2 a, 2 d, s2 c	KDH368	*MATa his3Δ1 leu2Δ0 met15Δ0 ura3Δ0 Ctf3-GFP::HIS3*
2 a, 2 d, s2 c	KDH370	*MATa his3Δ1 leu2Δ0 met15Δ0 ura3Δ0 Chl4-GFP::HIS3*
2 a, 2 d, s2 c, s3 a	KDH366	*MATa his3Δ1 leu2Δ0 met15Δ0 ura3Δ0 Ctf19-GFP::HIS3*
2 a, 2 d, 3 e, s2 c, s3 a	KDH324	*MATa his3Δ1 leu2Δ0 met15Δ0 ura3Δ0 Ame1-GFP::HIS3*
2 a, 2 d, 3 e, s2 c, s3 a	KDH325	*MATa his3Δ1 leu2Δ0 met15Δ0 ura3Δ0 Okp1-GFP::HIS3*
2 a, 2 d, 3 e, s2 c, s3 a	KDH367	*MATa his3Δ1 leu2Δ0 met15Δ0 ura3Δ0 Spc105-GFP::HIS3*
2 a, 2 d, s2 c, s3 a	KDH318	*MATa his3Δ1 leu2Δ0 met15Δ0 ura3Δ0 Cnn1-GFP::URA3*
2 b, 2 d, 3 e, s2 c, s3 b	KDH235	*MATa his3Δ1 leu2Δ0 met15Δ0 ura3Δ0 Mtw1-GFP::HIS3*
2 b, 2 d, 3 e, s2 c, s3 b	KDH371	*MATa his3Δ1 leu2Δ0 met15Δ0 ura3Δ0 Nnf1-GFP::HIS3*
2 b, 2 d, 3 e, s2 c, s3 b	KDH372	*MATa his3Δ1 leu2Δ0 met15Δ0 ura3Δ0 Nsl1-GFP::HIS3*
2 b, 2 d, 3 e, s1 b, s1 c, s2 a, s2 c	KDH179.1	*MATa his3Δ1 leu2Δ0 met15Δ0 ura3Δ0 Dsn1-GFP::URA3*
2 b, 2 d, 3 e, s2 c, s3 c	KDH374	*MATa his3Δ1 leu2Δ0 met15Δ0 ura3Δ0 Spc24-GFP::HIS3*
2 b, 2 d, 3 e, s2 c, s3 c	KDH373	*MATa his3Δ1 leu2Δ0 met15Δ0 ura3Δ0 Spc25-GFP::HIS3*
2 b, 2 d, 3 e, s2 c, s3 c	KDH180.1	*MATa his3Δ1 leu2Δ0 met15Δ0 ura3Δ0 Nuf2-GFP::URA3*
2 c, 2 d, 3 e, s2 c, s3 d	KDH233	*MATa his3Δ1 leu2Δ0 met15Δ0 ura3Δ0 Ask1-GFP::HIS3*
2 c, 2 d, 3 e, s2 c, s3 d	KDH375	*MATa his3Δ1 leu2Δ0 met15Δ0 ura3Δ0 Dd1-GFP::HIS3*
4 a, 4 b, 4 e, 4 f, 6 f	KDH199.1	*MATa his3Δ1 leu2Δ0 met15Δ0 ura3Δ0 Ndc80-tdEos::URA3*
4 c, 4 d, 4 e, 4 f	KDH226.1	*MATa his3Δ1 leu2Δ0 met15Δ0 ura3Δ0 Ask1-tdEos::URA3*
4 e, 4 f, s4 e	KDH229.1	*MATa his3Δ1 leu2Δ0 met15Δ0 ura3Δ0 Dm1-tdEos::URA3*
4 e, 4 f, s4 c, 5 a, 5 b, 5 c	KDH407.1	*MATa his3Δ1 leu2Δ0 met15Δ0 ura3Δ0 Dsn1-tdEos::URA3*
4 e, 4 f, s4 c	KDH220.1	*MATa his3Δ1 leu2Δ0 met15Δ0 ura3Δ0 Mtw1-tdEos::URA3*
s4 d	KDH277	*MATa his3Δ1 leu2Δ0 met15Δ0 ura3Δ0 Spc24-tdEos::URA3*
4 e, 4 f, s4 d	KDH223.1	*MATa his3Δ1 leu2Δ0 met15Δ0 ura3Δ0 Spc25-tdEos::URA3*
4 e, 4 f, s4 c	KDH198.1	*MATa his3Δ1 leu2Δ0 met15Δ0 ura3Δ0 Nsl1-tdEos::URA3*
4 e, 4 f, s4 b	KDH404.1	*MATa his3Δ1 leu2Δ0 met15Δ0 ura3Δ0 Spc105-tdEos::URA3*
s4 b	KDH217	*MATa his3Δ1 leu2Δ0 met15Δ0 ura3Δ0 Cnn1-tdEos::URA3*
6 a	KDH189.1	*MATa his3Δ1 leu2Δ0 met15Δ0 ura3Δ0 Nuf2-GFP::URA3,bik1Δ:: KANMX6*
6 a	MMA196	*MATa his3Δ1 leu2Δ0 met15Δ0 ura3Δ0 Nuf2-GFP::URA3,bim1Δ:: KANMX6*
6 a, 6 c, 6 d	KDH193.1	*MATa his3Δ1 leu2Δ0 met15Δ0 ura3Δ0 Nuf2-GFP::URA3,stu2-10:: KANMX6*
6 b	KDH538	*MATa his3Δ1 leu2Δ0 met15Δ0 ura3Δ0 Ask1-GFP::URA3,bik1Δ:: KANMX6*
6 b	KDH537	*MATa his3Δ1 leu2Δ0 met15Δ0 ura3Δ0 Ask1-GFP::URA3,bim1Δ:: KANMX6*
6 b	KDH399.1	*MATa his3Δ1 leu2Δ0 met15Δ0 ura3Δ0 Dm1-GFP::URA3,stu2-10:: KANMX6*
6 e, 6 f	KDH323.1	*MATa his3Δ1 leu2Δ0 met15Δ0 ura3Δ0 Ndc80-tdEos::URA3,stu2-10:: KANMX6*
7 a	MM197	*MATa his3Δ1 leu2Δ0 met15Δ0 ura3Δ0 Nuf2-GFP::URA3, mcm21Δ:: KANMX6*
7 a	MM198	*MATa his3Δ1 leu2Δ0 met15Δ0 ura3Δ0 Dsn1-GFP::URA3, mcm21Δ:: KANMX6*
7 b	KDH151	*MATa his3Δ1 leu2Δ0 met15Δ0 ura3Δ0 Nuf2-GFP::URA3, vik1Δ:: KANMX6*
7 b	KDH150	*MATa his3Δ1 leu2Δ0 met15Δ0 ura3Δ0 Dsn1-GFP::URA3, vik1Δ:: KANMX6*
7 b	KDH148	*MATa his3Δ1 leu2Δ0 met15Δ0 ura3Δ0 Nuf2-GFP::URA3,cik1Δ:: KANMX6*
7 b	KDH147	*MATa his3Δ1 leu2Δ0 met15Δ0 ura3Δ0 Dsn1-GFP::URA3,cik1Δ:: KANMX6*
7 c	KDH156	*MATa his3Δ1 leu2Δ0 met15Δ0 ura3Δ0 Dsn1-GFP::URA3,kip3Δ:: KANMX6*
7 c	KDH157	*MATa his3Δ1 leu2Δ0 met15Δ0 ura3Δ0 Nuf2-GFP::URA3,kip3Δ:: KANMX6*
8 a	KDH644	*MATa his3Δ1 leu2Δ0 met15Δ0 ura3Δ0 Nuf2-GFP::URA3,dam1-11:: KANMX6*
8 b	KDH143.1	*MATa his3Δ1 leu2Δ0 met15Δ0 ura3Δ0 Nuf2-GFP::URA3,cnn1Δ:: KANMX6*
8 c	KDH396	*MATalys2-801,ura3-52,leu2-3,112::Cnn1-16A-6xFlag::LEU2, Nuf2-GFP::URA3,Nnf1-3xHA:: KANMX*
8 c	KDH483	*MATa his3Δ1 leu2Δ0 met15Δ0 ura3Δ0 Nuf2-GFP::URA3,spc105-15:: KANMX6*
s5 a	KDH247	*MATa his3Δ1 leu2Δ0 met15Δ0 ura3Δ0 Nuf2-GFP::URA3,ipl1-2:: KANMX6*
s5 b	KDH379	*MATa his3Δ1 leu2Δ0 met15Δ0 ura3Δ0 Nuf2-GFP::URA3,cdc14-3:: KANMX6*
s5 c	KDH701	*MATa his3Δ1 leu2Δ0 met15Δ0 ura3Δ0 Nuf2-GFP::URA3,cdc11-4:: KANMX6*
s5 c	KDH702	*MATa his3Δ1 leu2Δ0 met15Δ0 ura3Δ0 Nuf2-GFP::URA3,cdc10-5:: KANMX6*
**Fission yeast (*S. pombe*)**		
9 d	KDH384	h^+ ade6-M210 leu1-32 ura4-D18 his3-D1 *Cnp1-GFP::NATMX6*
9 a, 9 d	KDH385	h^+ ade6-M210 leu1-32 ura4-D18 his3-D1 *Dsn1-GFP::NATMX6*
9 b, 9 c, 9 d	KDH386	h^+ ade6-M210 leu1-32 ura4-D18 his3-D1 *Ndc80-GFP::NATMX6*
9 d	KDH387	h^+ ade6-M210 leu1-32 ura4-D18 his3-D1 *Nuf2-GFP::NATMX6*

Table S2. **Comparison of kinetochore copy number in anaphase**

Complex	Protein	Anaphase number (Joglekar et al., 2006)	Anaphase number (this study)
Nucleosome	Cse4p	2	2
Cbf3	Ndc10p	2–3	3
	Mif2p	1–2	2
COMA	Ctf19p	2	3
	Spc105	5	5
MIND	Mtw1p	4–5	7
NDC80	Nuf2p	7	10
DAM-DASH	Ask1p	10–11	10
CTF3	Ctf3p	1	1
CHL4-IML3	Chl4p	<1	<1

File S1 is a separate zip file containing ImageJ macros and plugins used for this study.

Reference

Joglekar, A.P., D.C. Bouck, J.N. Molk, K.S. Bloom, and E.D. Salmon. 2006. Molecular architecture of a kinetochore-microtubule attachment site. *Nat. Cell Biol.* 8:581–585. http://dx.doi.org/10.1038/ncb1414
